# Comparative genomics and methylome profiling of Pseudolactococcus laudensis reveal signatures of niche adaptation and strain-level variation in mobile genetic elements and phage defence

**DOI:** 10.1099/mgen.0.001779

**Published:** 2026-07-06

**Authors:** Axel Soto-Serrano, Tamas Vincze, Richard J. Roberts, Lukasz Krych, Jennifer Mahony, Paulina Deptula

**Affiliations:** 1 Section for Food Microbiology, Gut Health and Fermentation, Department of Food Science, University of Copenhagen, Rolighedsvej 26, 1958 Frederiksberg C, Denmark; 2 New England Biolabs, Ipswich, MA 01938-2723, USA; 3 School of Microbiology & APC Microbiome Ireland, University College Cork, Cork, Ireland

**Keywords:** bacteriophage, defence, DNA methylation, horizontal gene transfer (HGT), laudensis, lactic acid bacteria (LAB), mobile genetic element (MGE), niche adaptation, phage, *Pseudolactococcus*

## Abstract

*Pseudolactococcus laudensis* (formerly named *Lactococcus laudensis*) is an emerging lactic acid bacterium first isolated from raw milk in 2015 and subsequently detected in vegetables and dairy mesophilic starter cultures. Despite its recurrent isolation from diverse environments, the genetic basis of its niche adaptation, horizontal gene transfer and phage defence remains unexplored. Here, we perform the first comparative genomic and epigenomic analysis of *P. laudensis* using complete genomes of a plant-derived isolate (MCRI-603), a milk isolate (DSM 28961) and 20 strains from a Danish dairy mesophilic starter culture. Genomes were annotated and analysed using pangenomics, Clustering of Orthologous Genes and methylome profiling. Average nucleotide identity, pangenome and Clustering of Orthologous Genes analyses revealed niche-associated structure: dairy starter strains formed a tight cluster, while the plant isolate MCRI-603 and milk isolate DSM 28961 were more similar to each other than to the starter culture group. The pangenome comprised 4,946 genes, with 1,396 core genes. Dairy starter strains showed markedly elevated numbers of insertion sequences, pseudogenes, plasmids and genomic islands relative to MCRI-603, which was plasmid-free and carried very few insertion sequence elements or genomic islands. DSM 28961 displayed pseudogene count similar to the dairy starter strains but markedly fewer transposases. These patterns are consistent with a plant-associated origin of *P. laudensis* and progressive dairy specialization via mobile genetic element acquisition. The *P. laudensis* mobilome was found to carry key niche-related traits. Lactose utilization operons were plasmid-encoded, whereas exopolysaccharide-encoding loci, *opp* oligopeptide transport systems and several defence loci, including clustered regularly interspaced short palindromic repeats and CRISPR-associated proteins (CRISPR-Cas), were consistently encoded within chromosomal integrative elements. All strains harboured prophage-like elements, including putatively intact prophages in 13 of them, and ~67% of 238 predicted antiphage systems resided on mobile genetic elements, underscoring their central role in phage defence. Restriction-modification systems dominated the defensome, and three strains encoded CRISPR-Cas systems (including type III-A and type I-C), indicating a higher prevalence than has been reported for *Lactococcus lactis* and *Lactococcus cremoris*, where CRISPR-Cas has rarely been observed. Methylome analysis identified 43 distinct motifs, of which 25 were novel. The *P. laudensis* methylome was overwhelmingly dominated by N⁶-methyladenine, and most motifs were short, non-palindromic and largely associated with type III restriction-modification systems and some type I and II subtypes. Nearly all strains exhibited distinct methylation profiles, including those isolated from the same dairy starter culture, highlighting extensive epigenetic diversification in dairy environments. Altogether, the data reveals a highly dynamic genomic and epigenomic landscape in *P. laudensis*, greatly shaped by mobile genetic elements, and provides a foundation for future work in this species and other Pseudolactococci.

**Impact Statement**

*Pseudolactococcus laudensis* is a lactic acid bacterium that is increasingly detected in dairy starter cultures but remains poorly characterized compared with model lactococci. This study provides the first comparative genomics and methylome profiling analysis of *P. laudensis* using all currently available complete genomes, including a plant isolate, a milk isolate and 20 strains from a mesophilic starter culture. By integrating pangenome, mobilome, defensome, prophage and DNA methylation analyses, we show that dairy-associated *P. laudensis* strains likely descend from plant-associated ancestors and display extensive genome plasticity shaped by mobile genetic elements, which carry key traits for dairy adaptation and bacteriophage resistance. We ascertain that functions that are often plasmid-borne in *Lactococcus*, such as peptide transport or exopolysaccharide loci, were frequently encoded in genomic islands in *P. laudensis* and also report highly strain-specific defensome, prophage presence and methylation profiles, even among strains isolated from the same dairy starter culture, highlighting epigenetic diversity with practical utility for biotechnological applications in dairy. Overall, this work advances understanding of this emerging bacterium and the below-species-level heterogeneity in dairy communities, while at the same time, it provides a framework for future studies in other species from the *Pseudolactococcus* genus.

## Data Summary

The authors confirm that all supporting data and protocols have been provided within the article or through supplementary data files. The accession numbers of the *Pseudolactococcus laudensis* assemblies utilized in this study are as follows: MCRI-603, CP199848; DSM 28961, CP186774–CP186776; and Tistrup MSC dairy strains: CP186777–CP186859.

## Introduction

Lactic acid bacteria (LAB) are central to food fermentations, with species of the genus *Lactococcus* playing a pivotal role in milk acidification, flavour formation and food safety. *Lactococcus lactis* and *Lactococcus cremoris* (formerly *L. lactis* subsp. *cremoris*) [1] have been widely used as starter cultures and are considered model organisms for food fermentations [2].

Until recently, 26 lactococcal species were described, of which 17 have been isolated within the last decade from several niches, including red meat [3], goat’s milk [4], kimchi [5], the facial abscess of a marsupial [6], the guts of mice [7,8] and various insects such as beetles or termites [9,13], among others. Recently (June 2025), phylogenetic differences among *Lactococcus* species resulted in the reclassification of the genus, which is now represented by two genera, namely, *Lactococcus* and *Pseudolactococcus* [14].

Only five lactococcal or pseudolactococcal species are described to have been isolated from milk or dairy environments, namely, the aforementioned *L. lactis* and *L. cremoris*, as well as *Lactococcus hircilactis*, *Pseudolactococcus raffinolactis* and *Pseudolactococcus laudensis*. However, the ecological roles and potential industrial relevance, especially of *Pseudolactococcus* in microbial starter cultures, remain poorly understood [15].

Notably, dairy lactococci are believed to have evolved from plant-associated isolates [16,21]. The dairy environment is characterized by stable, nutrient-rich conditions and reduced selective pressure for metabolic versatility [22]. Thus, this ecological transition promoted a process of reductive evolution resulting in the loss of genes required for the degradation of complex plant polymers and the parallel acquisition or retention of traits advantageous for milk fermentation, such as lactose metabolism genes and enhanced acidification as well as proteolytic and peptidolytic capacities [2]. This niche adaptation in lactococci is largely attributable to an accessory genome enriched in mobile genetic elements (MGEs) [23,24] such as plasmids, genomic islands (GIs) and prophages, also known as their mobilome. These MGEs facilitate horizontal gene transfer (HGT) and carry essential genes that enable lactococci to thrive in dairy environments, including those involved in lactose and citrate metabolism [25], as well as diverse bacteriophage (phage) defence mechanisms that are crucial in industrial applications [26,27], among others.

While extensive research has focused on the well-characterized species *L. lactis* and *L. cremoris*, emerging species like *P. laudensis* remain underexplored despite their presence in diverse ecological niches, including raw milk [4], plant environments [28] and multiple dairy starter cultures [29,31]. Recently, the first complete *P. laudensis* genomes were released, comprising 20 strains isolated from a single Danish mesophilic starter culture (MSC) and one cow milk isolate from Italy [32].

In the current study, we obtained the complete genome of the only *P. laudensis* plant isolate known to date, MCRI-603 [28], and performed the first comprehensive comparative genomic analysis of the *Pseudolactococcus* genus using this and all the complete genomes from Soto-Serrano *et al*. [32]. Our work focuses on elucidating their genetic composition, providing insights into their mobilome, phage receptors and defence systems, as well as other traits relevant for industrial applications and niche adaptation. Furthermore, we resolve the methylomes of the studied isolates and, where possible, link methylated motifs to their cognate restriction–modification (RM) genes. The data presented here provide an additional functional layer that can be exploited in dairy fermentations for strain selection, starter culture design and improved robustness against phages. Finally, this work highlights similarities and differences between dairy *Lactococcus* and *Pseudolactococcus*, laying the foundation for future work on the latter.

## Methods

### DNA sequencing and genome assembly

*P. laudensis* MCRI-603 was cultivated on MRS agar (Oxoid, Hampshire, UK) at 30 °C for 3 days under anaerobic conditions. Genomic DNA was extracted from colonies using the Genomic Tip 20/G (#10223) in combination with Genomic DNA Buffer Set (#19060) (QIAGEN, Tokyo, Japan) following the manufacturer’s protocol for Gram-positive bacteria. Purified genomic DNA was provided by Marudai Food Co., Ltd. (Osaka, Japan).

Library preparation was performed using Native Barcoding Sequencing protocol (SQK-NBD114.96), previously normalizing the DNA concentration to 33.33 ng µl^−1^ (400 ng total). The sequencing was performed on a PromethION 2 Solo platform (Oxford Nanopore Technologies, Oxford, UK) and basecalled with Dorado v0.9.1 (https://github.com/nanoporetech/dorado) with the dna_r10.4.1_e8.2_400bps_sup@v5.0.0 basecalling model.

Raw data was filtered using NanoFilt v2.6.0 [33] to a minimum quality of 8 and a minimum length of 1,000 bp. Assembly was performed utilizing Trycycler v0.5.4 [34] with the data obtained after dividing the reads into 12 subsets and assembling those with Flye v2.9.1 [35], Canu v2.2 [36] and Hybracter v0.9.0 [37]. The genome was reoriented to start from *dnaA* using dnaapler v0.8.1 [38].

The genomes of the DSM 28961 and Tistrup MSC strains were obtained from [32]. The NCBI accession numbers of the *P. laudensis* assemblies utilized in this study are as follows: MCRI-603, CP199848; DSM 28961, CP186774–CP186776; and Tistrup MSC dairy strains, CP186777–CP186859. Quality of the genomes was assessed using CheckM v1.2.4 [39].

### Average nucleotide identity, gene annotation and pangenome analysis

Average nucleotide identity (ANI) analysis was performed using Skani v0.2.2 [40]. For pangenome analysis, gene annotation was performed using Bakta v1.9.3 using the v5.1 database [41], and the obtained output was utilized as input for further analysis with Roary v3.13.0 [42] with default settings. Roary results were visualized with Phandango v1.3.1 [43]. Pangenome openness was assessed using the R package micropan v1.2 [44], with Heaps’ law analysis performed using 10,000 random genome-addition permutations and a fixed random seed for reproducibility. The fitted Heaps’ law exponent (*α*) was used to classify the pangenome as open (*α*<1) or closed (>1). The core phylogenetic tree was created using FastTree v2.1.11 [45]. This and other phylogenetic trees in the analysis were visualized with the Interactive Tree of Life (iTOL) v7 [46].

Clusters of Orthologous Groups (COGs) were classified with COGclassifier v2.0.0 (https://github.com/moshi4/COGclassifier) and visualized using Vega-Lite v6.1.0 (https://vega.github.io/vega-lite). Genes encoding antimicrobial compounds resistance were determined using ResFinder v4.5.0 [47], using the 2.3.1 database with default settings, as well as CARD RGI v6.0.5 [48] with default settings.

### Prediction of plasmid mobility, host range and replication type

Prediction of plasmid mobility and host range was performed with MOB-suite v3.1.9 [49], using mob_typer with the full circularized contigs as an input and utilizing the -x flag. Plasmid contigs were further analysed with OriTfinder2 v2.0 [50]. Plasmid replication type was determined as described previously [26]. Briefly, plasmids whose *repB* gene was preceded by 3.5–4 iterations of a 22 bp tandem repeat, which at the same time was preceded by an A/T rich 10 bp imperfect repeat further upstream, were considered to possess a Theta replication mode [51]. Tandem repeats were determined using Tandem Repeats Finder v4.02 [52] with the ‘show flanking regions’ function to manually inspect the upstream sequence in order to find the A/T rich 10 bp imperfect repeat.

### Prediction and analysis of prophages, phage satellites and other GIs

GIs were predicted and visualized using IslandCompare v1.1 [53]. Prophage regions were predicted and annotated using Phastest v3.0 [54]. ICEfinder from ICEBerg 3.0 [55] and OriTfinder2 v2.0 [50] were used to predict origin of transfer (OriT), relaxases, type IV secretion systems (T4SS) and type IV coupling protein (T4CP) encoding genes. Additional HGT regions were surveyed with Alien Hunter v1.7 [56] via the Proksee webserver [57]. Arrow plots for representation and visualization of specific gene clusters in the present study were generated with Clinker [58] via the CAGECAT webserver, release 1.0 [59].

Phage satellites were identified using SatelliteFinder v0.9.1 [60], implemented via MacSyFinder v2.0 [61]. Each strain was analysed independently against all four phage-inducible chromosomal island (PICI) family models (PICI, P4, PLE and cfPICI) using --db-type ordered_replicon. To compare PICI gene content across strains, protein sequences from all detected PICI systems were pooled and clustered using CD-HIT v4.8.1 [62] at 40% identity and 70% alignment coverage of the longer sequence (-c 0.4 -aL 0.7 n 2). The resulting protein clusters were used to construct a binary presence/absence matrix, from which pairwise Jaccard distances were calculated. PICI systems were grouped into backbone types by average-linkage hierarchical clustering using SciPy v1.13.1 [63], with a Jaccard distance threshold of 0.4. Within each backbone group, PICI systems were further ordered by hierarchical clustering on their gene presence/absence profile. For each backbone group, the PICI system with the greatest number of detected genes was designated as the reference. Protein sequence identity to the reference was calculated for each gene using Basic Local Alignment Search Tool (blastp) v2.15.0+ [64].

### Prediction of bacteriophage defence systems and DNA methylation analysis

Bacteriophage defence systems were predicted employing PADLOC v2.0.0 [65] and DefenseFinder v2.0.0 [66]. Furthermore, blastp [64] was utilized for the identification of antiphage systems recently discovered in the lactococcal plasmidome [67].

Superinfection exclusion (Sie) systems were predicted as described previously [68,69]: candidates of Sie-encoding genes were selected based on their genomic location within the lysogeny module of prophages, i.e. between the integrase and the repressor genes. To be considered Sie, the genes had to possess one or more N-terminal transmembrane domain(s), which were predicted with DeepTMHMM v1.0 [70].

To perform DNA methylation analysis, the modified base models dna_r10.4.1_e8.2_400bps_sup@v5.0.0_6mA@v3 and dna_r10.4.1_e8.2_400bps_sup@v5.0.0_4mC_5mC@v3 were utilized during basecalling. Methylated motifs were determined with Nanomotif v0.5.8 [71], following Nanomotif’s documentation (https://nanomotif.readthedocs.io/en/latest/index.html) and using nanomotif find_motifs. Association of specific motifs to RM systems or orphan methyltransferases (MTases) was performed essentially as previously described [72]. Briefly, RM systems were identified using the SEQWARE computer resource [72], a blast-based software module in combination with the curated restriction enzyme database (REBASE) [73]. Prediction was supported by sequence similarity, presence and order of predictive functional motifs, in addition to the known genomic context and characteristics of empirically characterized RM system genes within REBASE and enabled the reliable assignment of candidate MTase genes to each specificity based on their RM types.

## Results

### General features and pangenome analysis

The complete genome of the *P. laudensis* MCRI-603 was sequenced and assembled in this study and analysed alongside the first previously reported complete genomes of *P. laudensis* [32]. In total, this dataset comprises 22 strains isolated from bovine milk (DSM 28961) [4], kale (MCRI-603) [28], and the ‘Tistrup’ dairy MSC (all remaining strains) [29,32], representing the full set of complete genomes currently available for this species. CheckM analysis showed high completeness (97.36–98.11%) and low contamination (0.25–1.19%) for all genomes, supporting assembly quality (Table S1, available in the online Supplementary Material).

On average, *P. laudensis* genomes have a G+C content of 38.62 mol% and a size of 2.379 Mbp, with strain-specific lengths ranging from 2,330,094 bp (T2A6) to 2,420,405 bp (T2H4), chromosome sizes from 2,216,492 bp (T2F2) to 2,351,550 bp (T2A7) and a variable plasmid count from none (MCRI-603) to five (T2F2). Thus, a higher number of plasmids is present in dairy MSC *P. laudensis* than in its plant counterparts, similar to what has been reported in *L. cremoris* and *L. lactis* [26]. However, dairy *P. laudensis* appear to harbour comparatively fewer plasmids than, e.g. dairy *L. lactis*, which can carry up to 12 [25]. In *P. laudensis*, the plasmidome constitutes, on average, 3.78% of the total genome, reaching 6.57% in some dairy strains such as T2C6 (Table 1).

**Table 1.** General genomic features of the 22 *P*. *laudensis* strains

Strain name	Genome size (bp)	GC content (mol%)	Chromosome size (bp)	Plasmidome size (bp)	% of the genome	# plasmids	Isolation source	Country	Accession numbers
T2A4	2,398,886	38.53	2,283,261	115,625	4.82%	3	MSC	Denmark	CP186777–CP186780
T2A6	2,330,094	38.62	2,217,664	112,430	4.83%	4	MSC	Denmark	CP186781–CP186785
T2A7	2,402,460	38.55	2,351,550	50,910	2.12%	2	MSC	Denmark	CP186786–CP186788
T2A8	2,385,833	38.56	2,283,490	102,343	4.29%	3	MSC	Denmark	CP186789–CP186792
T2C1	2,386,128	38.65	2,288,839	97,289	4.08%	3	MSC	Denmark	CP186793–CP186796
T2C6	2,372,414	38.54	2,216,499	155,915	6.57%	4	MSC	Denmark	CP186797–CP186801
T2C9	2,367,517	38.62	2,308,210	59,307	2.51%	3	MSC	Denmark	CP186802–CP186805
T2D8	2,398,755	38.59	2,338,203	60,552	2.52%	3	MSC	Denmark	CP186806–CP186809
T2E11	2,377,185	38.64	2,282,564	94,621	3.98%	4	MSC	Denmark	CP186810–CP186814
T2E12	2,359,441	38.57	2,216,499	142,942	6.06%	4	MSC	Denmark	CP186815–CP186819
T2E8	2,388,398	38.62	2,337,488	50,910	2.13%	2	MSC	Denmark	CP186820–CP186822
T2F10	2,386,112	38.58	2,253,378	132,734	5.56%	3	MSC	Denmark	CP186823–CP186826
T2F2	2,339,063	38.61	2,216,492	122,571	5.24%	5	MSC	Denmark	CP186827–CP186832
T2F8	2,387,940	38.61	2,337,031	50,909	2.13%	2	MSC	Denmark	CP186833–CP186835
T2G11	2,383,214	38.64	2,337,423	45,791	1.92%	2	MSC	Denmark	CP186836–CP186838
T2G3	2,389,112	38.62	2,285,454	103,658	4.34%	3	MSC	Denmark	CP186839–CP186842
T2G5	2,412,245	38.53	2,317,268	94,977	3.94%	3	MSC	Denmark	CP186843–CP186846
T2H1	2,372,941	38.62	2,271,033	101,908	4.29%	3	MSC	Denmark	CP186847–CP186850
T2H3	2,362,299	38.66	2,294,352	67,947	2.88%	4	MSC	Denmark	CP186851–CP186855
T2H4	2,420,405	38.51	2,317,283	103,122	4.26%	3	MSC	Denmark	CP186856–CP186859
DSM28961	2,399,702	38.69	2,285,465	114,237	4.76%	2	Cow milk	Italy	CP186774–CP186776
MCRI-603	2,316,755	39.2	2,316,755	0	0%	0	Kale	Japan	CP199848

**Fig. 1.**
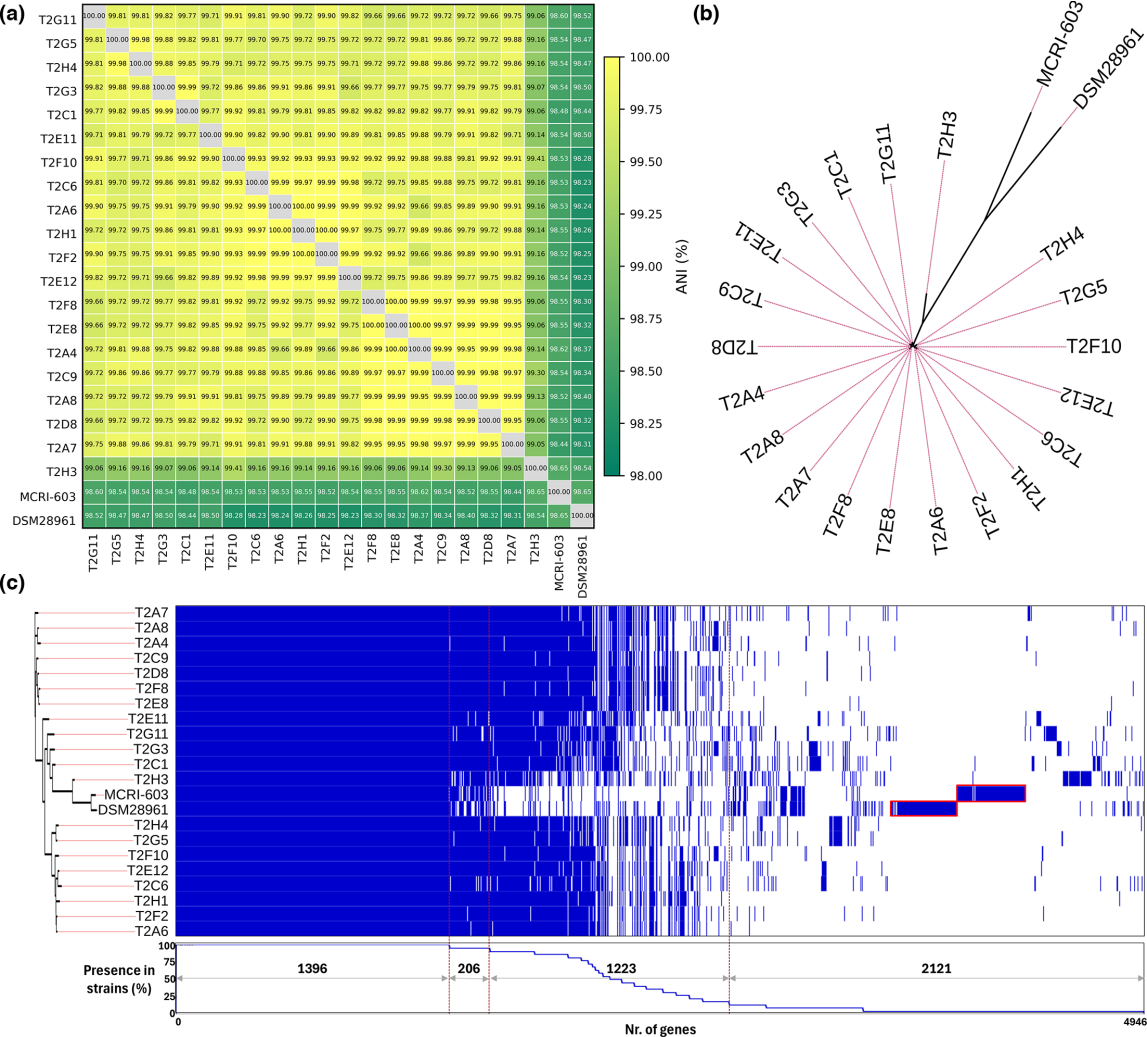
Genome relatedness and pangenome structure in *P. laudensis*. (**a**) Pairwise ANI heatmap with hierarchical clustering (tree not shown). The colour scale indicates ANI (%). (**b**) Unrooted core-genome phylogeny inferred from the concatenated alignment of the 1,396 single-copy core genes. (**c**) Pangenome gene presence/absence matrix; rows correspond to strains and columns to a total of 4,946 genes); filled cells indicate presence, and the phylogenetic tree is based on the presence/absence of accessory genes. Gene families framed in red unique to MCRI-603 and DSM 28961 mostly belong to mobilome-related functions (e.g. prophage genes and transposases), ABC transporters and genes for the biosynthesis of polysaccharides, presumably associated with the cell surface.

ANI among the dairy MSC strains was >99.66%, except for T2H3, with values ranging from 99.05 to 99.41%. By contrast, the MSC strains were more distant from the DSM 28961 and the MCRI-603 strain, with ANI values ranging from 98.23 to 98.54% and 98.44 to 98.65%, respectively (Fig. 1a). MCRI-603 and DSM 28961 were more similar between (ANI 98.65%) each other than they were to the MSC strains, strain T2H3 being the most similar. Overall, these genetic differences are consistent with the distinct geographical and ecological origins of the isolates (Table 1).

**Fig. 2.**
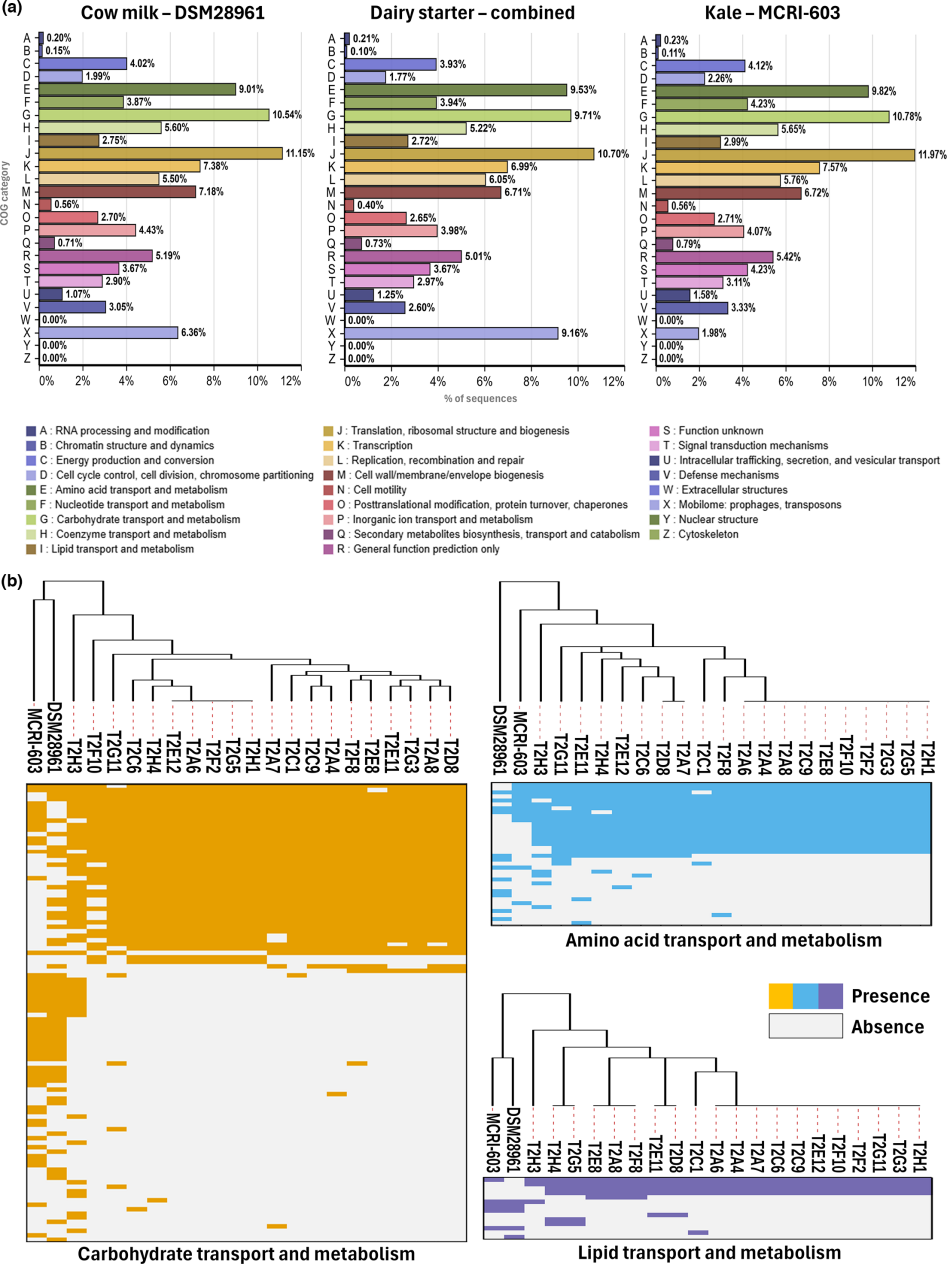
COG-based niche differentiation in *P. laudensis*. (**a**) Distribution of COG functional categories across strains; values denote the percentage of coding sequences assigned by COGclassifier. (**b**) Presence/absence matrix for non-redundant genes from COG [G] carbohydrate transport/metabolism (orange), COG [E] amino acid transport/metabolism (sky blue), and COG [I] lipid transport/metabolism (purple); only genes not present in all strains are shown. Orange (left), carbohydrates (G family); sky blue (top-right), amino acids (E) family; purple (bottom-right), lipids (I family). A complete list of COG annotations per strain is provided in Supplementary Data.

Similarly, pangenome analysis revealed that DSM 28961 and MCRI-603 are the most dissimilar strains in both accessory gene content and core genome sequence similarity, while the MSC strains clustered together (Fig. 2b, c). Because the latter isolates were all obtained from the same culture, this likely explains much of their phylogenetic similarity; however, previous work revealed substantial strain-level differences in loci encoding the biosynthesis of surface-associated polysaccharides, particularly exopolysaccharides (EPSs) [32]. Collectively, the analysis revealed an open *P. laudensis* pangenome (*α*=0.715) so far composed of 4,946 genes, of which 1,396 constitute the core genome (present in ≥99% of strains) and 206 the soft-core genome (95–98.9% of the strains), and the remainder are shell or cloud genes (<95% of the strains) (Fig. 1c), of which 1,438 genes were unique to single strains.

### Analysis of COGs

To investigate niche-specific adaptations in *P. laudensis*, we analysed the distribution of COG functional categories across all strains. Approximately 80% of coding sequences (CDSs) were assigned by COGclassifier. Overall, similar proportions were observed across most COG categories irrespective of niche, with the notable exception of category X (mobilome: transposons and prophages) (Fig. 2a), which accounted for 9.16%, 6.36% and 1.98% of classified COGs in dairy MSC, bovine milk and kale-derived strains, respectively.

In addition to the mobilome, the COG families G (carbohydrate transport and metabolism), E (amino acid transport and metabolism) and I (lipid transport and metabolism) are central for niche adaptation, as these determine the ability of the bacteria to metabolize key substrates. We generated presence/absence matrices for non-redundant genes within these categories to assess clustering by niche, as previously reported for *L. lactis*/*cremoris* [24]. The resulting patterns showed niche-associated structure: the dairy starter strains clustered together, whereas DSM 28961 (milk) and MCRI-603 (kale) each formed distinct, non-dairy branches. Although they did not cluster together, they were more similar to each other than to the dairy starter set. Among the dairy MSC strains, T2H3 was the closest to both, consistent with the overall gene presence/absence profiles, ANI and the core-genome phylogeny (Fig. 1). Across the three COG categories, carbohydrate transport/metabolism contributed the greatest diversity (103 variable genes not present in all strains), followed by amino acid (28) and lipid transport/metabolism (14) genes (Fig. 2a).

The most notable differences in carbohydrate transport and metabolism (Fig. 2b) were that the MSC and DSM 28961 strains harbour the *lac* operon associated with lactose utilization, whereas MCRI-603 did not, consistent with lactose being the principal carbon source in milk and highlighting metabolic adaptation to the dairy environment. DSM 28961 and the dairy MSC strains also encoded a *β*-xylosidase, while MCRI-603 lacked this function, despite *β*-xylosidases being central to the degradation of plant cell wall-derived lignocellulosic substrates [74]. Ribose ABC permeases were detected only in DSM 28961 and MCRI-603, whereas galactitol utilization genes were unique to MCRI-603. Although direct evidence for galactitol in kale is limited, galactitol is a common plant polyol [75].

With respect to amino acid transport and metabolism (Fig. 2b), all strains’ genomes possessed a conserved set of peptidases (PepN, PepD2, PepO, PepP, PepE, PepF and PepC), and all encoded oligo/dipeptide transport systems. The dairy MSC strains, however, carried the highest number, with ten peptide transport components per genome on average, including two sets of *opp* genes and *dppB*, crucial for peptide uptake and utilization in the dairy environment [76,77], while DSM 28961 and MCRI-603 encoded a single set of Opp transporters.

Examples of differences in lipid transport and metabolism genes (Fig. 2b) include enzymes annotated as gluconate 5-dehydrogenase in MCRI-603 and DSM 28961 and an (S)-acetoin-forming diacetyl reductase in the dairy MSC strains T2G5 and T2H4. Acetoin and especially diacetyl are aroma compounds providing a buttery flavour which can be desirable in certain cheeses [78]. Consequently, enzymatic reduction of diacetyl to acetoin may decrease the intensity of buttery flavour and thereby influence the sensory profile of the final product.

Given the clear mobilome differences across niches and its central role in dairy adaptation, where numerous carbohydrate/peptide transport functions and even phage-defence systems are MGE-encoded [69,79], the *P. laudensis* mobilome is examined in detail in the following sections, including the composition, distribution and overlap of plasmids, GIs and prophages.

### Transposases, genome decay and genome synteny

Transposases facilitate the movement of discrete DNA segments, so-called transposons, between genomic locations by catalysing DNA cleavage and rejoining [80]. This intrinsic mobility enables the spread of transposable elements within and across genomes, generating genetic mutations and rearrangements that promote biological diversification and evolution, ultimately making transposases the most abundant genes in nature [81]. Because transposition can truncate or disrupt genes, elevated counts of both pseudogenes and transposable elements, e.g. insertion sequences (ISs), are commonly taken as signatures of genome decay [24]. We therefore quantified these features across the *P. laudensis* genomes.

On average, the dairy MSC strains harboured 160.8 pseudogenes, ranging from 131 to 223 in T2F10 and T2C1, respectively. The bovine milk isolate DSM 28961 contained 153 pseudogenes, comparable to the MSC isolates despite its distinct (though related) ecological origin. In contrast, the plant isolate MCRI-603 exhibited the lowest levels of pseudogenes, with only 12 pseudogenes. Transposable element content also differed by niche: the dairy MSC isolates averaged 158.8 IS/transposase genes, reaching up to 174 in T2H4, while DSM 28961 encoded 107. In DSM 28961, the most abundant transposase families were IS3, IS5 and IS6 with 36, 25 and 13 genes, respectively, whereas in the MSC isolates IS5 elements (mostly annotated as IS1194) dominated, with an average of 79 genes per genome, followed by IS3 with 34.5 genes on average. Lastly, MCRI-603 harboured only two IS/transposase genes.

These trends mirror observations in *Lactococcus*: *L. cremoris* harbours far more pseudogenes (~100 vs. 31) and IS/transposase genes (~138 vs. 41) than *L. lactis* [24]. These counts are thought to reflect genome decay associated with prolonged adaptation to the dairy niche, from which most *L. cremoris* were isolated, in contrast to more diverse ecological niches of *L. lactis* isolates. Overall, our findings in *P. laudensis* are consistent with this pattern, suggesting a parallel trajectory of genome streamlining among milk/dairy-adapted *P. laudensis*. The minimal genome decay in MCRI-603 suggests a plant origin for the species.

**Fig. 3.**
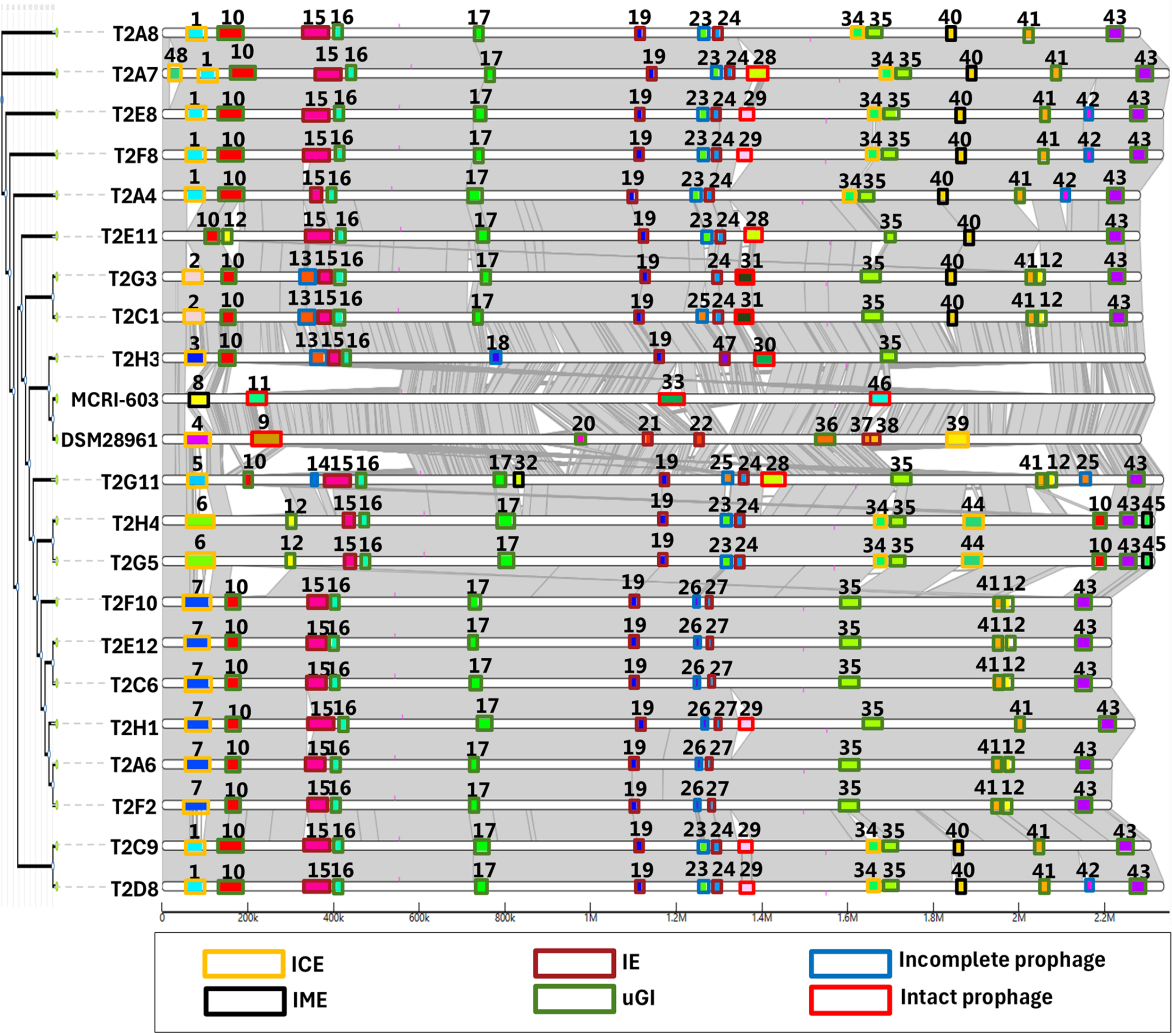
GIs and genome synteny. Boxes represent predicted GIs along each genome. Each GI cluster is indicated by a unique number and associated fill colour; therefore, islands with the same number and colour across different strains represent the same GI cluster. Box borders indicate the predicted MGE class (legend). ICE, integrative and conjugative element; IME, integrative and mobilizable element; IE, other integrative elements; uGI, unidentified genomic island.

Despite documented genomic rearrangements in key loci such as the *cwps* cluster in strains T2H4 and T2G5 [32], overall genome synteny was conserved among the dairy MSC isolates. In contrast, notable synteny disruption was observed between these strains and DSM 28961 and MCRI-603, which retained synteny with each other (Fig. 3).

### Plasmid mobility and niche adaptation

**Fig. 4.**
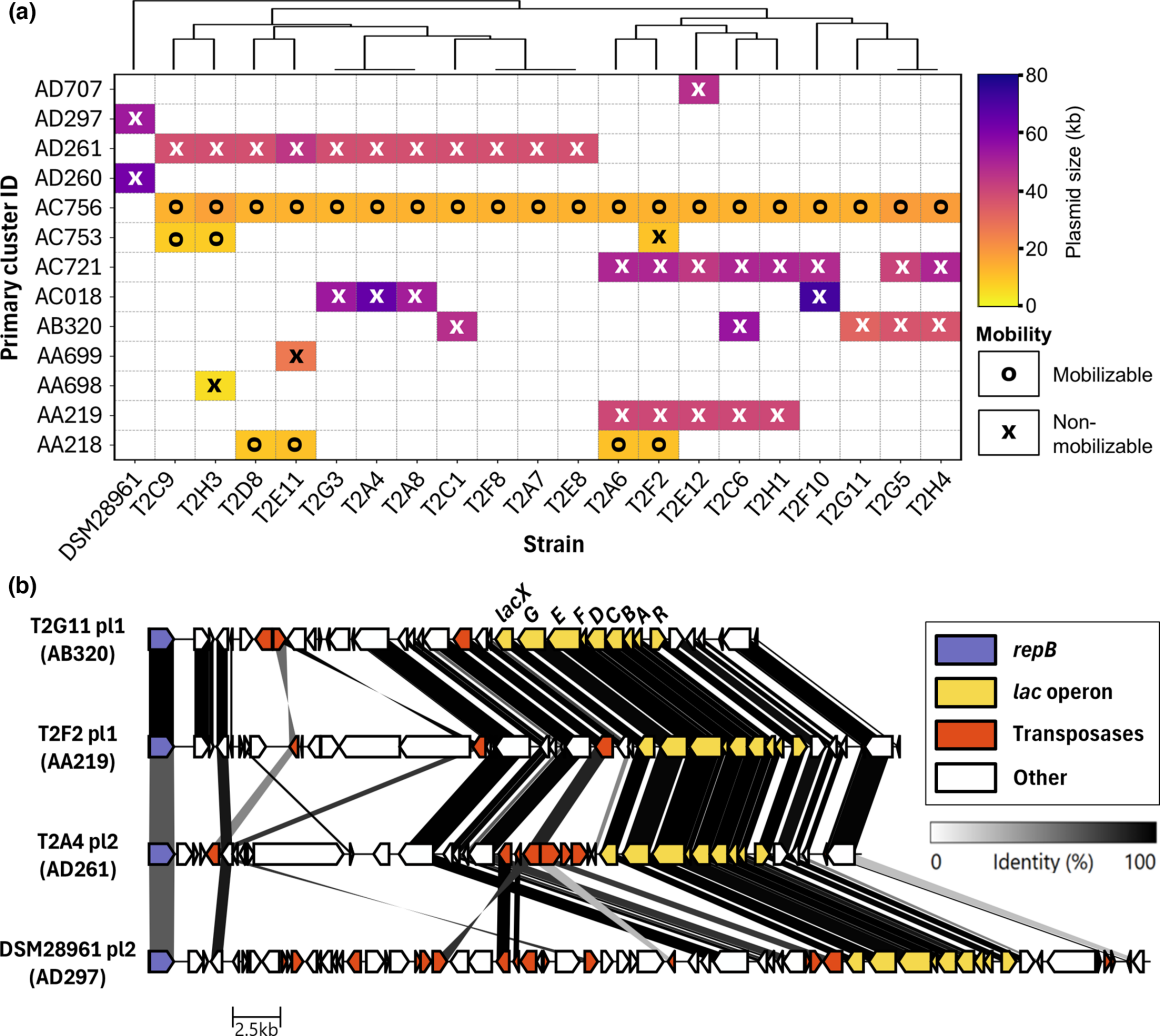
The *P. laudensis* plasmidome. (**a**) Presence/absence of the different plasmid clusters (based on MOB-suite cluster IDs), plasmid sizes and prediction of mobility. (**b**) Schematic depicting representatives of the different plasmid clusters containing a *lac* operon. Plasmid cluster IDs are indicated in parentheses.

Plasmids are self-replicating extrachromosomal elements of variable sizes that are essential for bacterial fitness and niche adaptation by mediating HGT of beneficial traits [82]. In the studied *P. laudensis* strains, plasmid sizes ranged from 5,140 bp to 72,676 bp in T2H3 plasmid 3 and T2F10 plasmid 1, respectively (Fig. 4a, Table S2). Plasmids were typed as described in Methods and are referred to by their MOB-suite cluster IDs to facilitate tracking and future comparisons. The 65 plasmids were classified into 13 cluster IDs; the most prevalent were AC756 (present in 20/21 strains), AD261 (11/21) and AC721 (8/21) (Fig. 4a).

Collectively, the 65 plasmids identified across the 21 milk and MSC genomes encode a total of 1,872 CDSs, of which 21.05% could not be annotated (annotated as hypothetical proteins), while the remaining 78.95% were either fully annotated or partially annotated, i.e. some information could be obtained, and these were not annotated as hypothetical proteins. Annotated genes present in the different plasmids are listed in Table S2. Furthermore, COG categories were assigned for the different plasmid clusters (Fig. S1).

In dairy lactococcal species including *L. cremoris* and *L. lactis*, key traits essential for growth in milk have often been observed to be plasmid-encoded, playing a central role in niche adaptation [25]. Notable examples include the caseinolytic cell-envelope proteinase (PrtP), the Opp oligopeptide transport system, the *citQRP* operon for citrate uptake and metabolism and the *lac* operon for lactose utilization [26,79]. In *P. laudensis*, *prtP* was absent from all strains, indicating dependence on accompanying microbiota for casein proteolysis and peptide supply. This was supported by the inability of the strains to grow in milk-based medium alone. Likewise, no *opp* or *citQRP* genes were detected on any of the plasmids analysed. Consistent with the absence of *citQRP*, growth of *P. laudensis* on KCA Leesment media indicated no citrate utilization. *P. laudensis* DSM 28961 has been previously tested for diacetyl production with positive results [83], but the lack of citrate utilization indicates an alternative pathway to citrate, likely alanine or aspartate catabolism in the presence of *α*-ketoglutarate [84].

By contrast, the 20 MSC strains and DSM 28961 harbour plasmids encoding a *lac* operon; interestingly, T2C1 encompasses it in both T2C1 plasmids 1 and 2. The plasmid clusters AB320 (except T2C6 plasmid 1), AA219, AD261 and AD297 carried identical *lac* operons embedded in distinct backbones [Figs 4 and S1; COG category (G)]. The *lac* regions were flanked by transposase genes, consistent with mobilization as transposable elements, as previously observed for Tn951 in *Yersinia enterocolitica* [85] and for lactose plasmids in *L. cremoris* SK11 and *L. lactis* ML3, where ISS1 elements flank *lac*, suggesting its mobilization as a composite transposon [86,87].

The dissemination of such adaptive traits through HGT is fundamental to niche specialization. Plasmids can be transferred from cell to cell via different mechanisms. In *L. lactis/cremoris*, plasmid transfer occurs mainly via conjugation and transduction [79]. For conjugation to occur, a full conjugation machinery is required, i.e. OriT, relaxase, T4SS and T4CP [88]. Non-conjugative plasmids can nonetheless be mobilized or co-mobilized together with conjugative plasmids if they encode at least an OriT [49,88, 89].

Of the 65 *P*. *laudensis* plasmids, 38 (58.46%) were classified as non-mobilizable, whereas 27 (41.54%) were predicted to be mobilizable, based on the absence or presence of a predicted OriT sequence, respectively (Fig. 4a, Table S2). Although some plasmids harbour genes associated with conjugation, none were predicted to form mating pores, preventing their self-transmission. Furthermore, none of the mobilizable plasmids exhibited >30% nucleotide similarity with the well-characterized lactococcal conjugation operons present in the pNP40 and pMRC101 plasmids [90,92]. Thus, none of the mobilizable plasmids were classified as conjugative. The plasmids were further scrutinized to find auxiliary genes of the relaxosome such as *mobC*, which have been shown to facilitate (co-)mobilization [90]. Only the mobilizable plasmid clusters AC756 and AC753 were found to harbour *mobC* genes (Table S2). Finally, all 65 plasmids were predicted to exhibit a theta replication system, in agreement with previous observations that theta replication predominates among lactococcal plasmids [26].

### Prophages and other GIs

GIs are distinct large regions of DNA (~10–200 kb) typically integrated into the chromosome which exhibit evidence of historical or current mobility. GIs may differ even among closely related bacterial strains, contributing to genome plasticity, facilitating adaptation and evolution [93,94]. GIs encompass diverse MGEs differing in structure and genetic composition, including integrative and mobilizable elements (IMEs), integrative and conjugative elements (ICEs), integrative elements (IE), transposons, integrated plasmids and prophages [95,96].

**Fig. 5.**
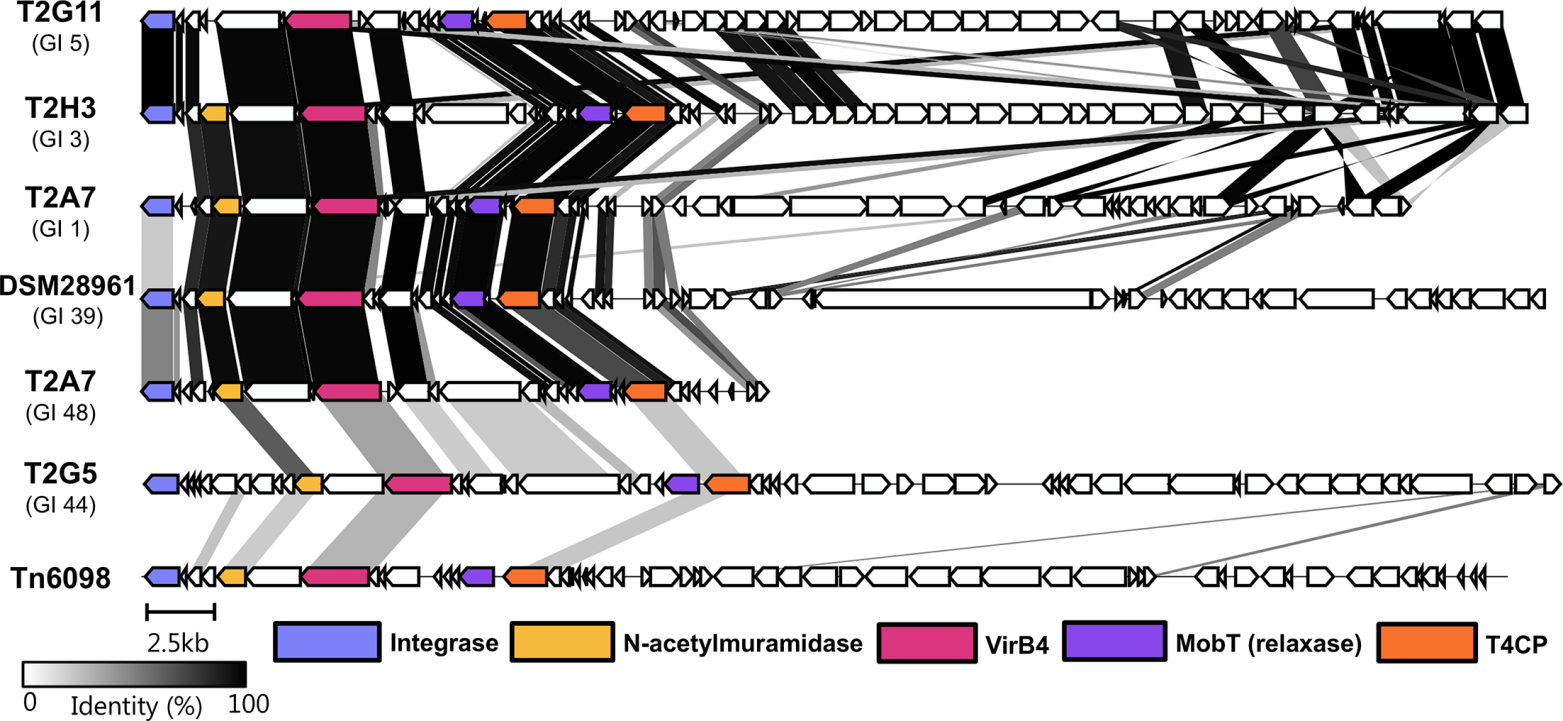
ICEs of *P. laudensis* compared to the previously characterized Tn6098. Coloured genes represent conserved ICE functions. GI numbers in parentheses correspond to the numbers in Fig. 3.

Among the *P. laudensis* strains, MCRI-603 harboured the fewest GIs (4), whereas T2G11 carried the most (15). GI sizes ranged from 8,055 to 65,830 bp (mean 24,248 bp), constituting ~9.75% of the chromosomal content of the species (Fig. 3). The GIs were further characterized and classified into the groups described by Audrey *et al*. [97]: unidentified GI (uGIs) (no annotated integrase at GI termini), IEs (integrase present), IMEs (integrase plus≥1 T4CP component) and ICEs (presence of a complete conjugation machinery). Prophages were assigned based on PHASTEST predictions and/or a majority of genes annotated as ‘phage protein’ by Bakta. The most frequent type of GI was uGIs (41.83%), followed by IEs (24.33%), prophages (16.35%), ICEs (12.17%) and IMEs (1.02%). ICEs were further inspected and conserved gene synteny was observed for the five core ICE functions, i.e. integrase, relaxase, *N*-acetylmuramidase, T4CP and the key T4SS component VirB4, consistent with *L. lactis* ICE architecture [98] (Fig. 5). OriTfinder2 was used to search for OriT sequences essential for self-transmission [99]; however, no ICE encoded a detectable OriT.

Beyond mobility-associated genes, GIs frequently carry cargo genes conferring adaptive advantages for their hosts [100]. For example, the ICE islands 1–7 (Fig. 3) carry genes encoding EPS production [32], and several GIs encode genes for phage defence systems, which will be described in the next subsection. All 20 starter-culture strains carry IE cluster 15, encoding the endopeptidase PepO and the oligopeptide transporter genes *oppABCDF*. As noted earlier, Opp systems are essential for growth in milk and are typically plasmid-encoded. Notably, blastn showed >99% identity to *L. lactis/L. cremoris* plasmid-borne *opp* loci, underscoring their mobility; conversely, the aforementioned second set of *opp* genes is also chromosomally encoded, seems to be conserved among all studied *P. laudensis* and is not embedded in an MGE. The IE 15 also encodes a type IV RM system.

In DSM 28961, GI clusters 36 and 37 encode genes for cadmium and arsenite resistance. The *cadA* gene did not present significant amino acid homology with that of the lactococcal plasmid pNP40 [92], but blastp revealed similarity (>95%) with heavy metal translocating ATPases from other *Pseudolactococcus* and non-dairy *Streptococcus*. Additionally, the uGI cluster 36 contains type II and type IV RM systems, *accABCD* and *fabZFGDKH* operons for fatty acid biosynthesis and a serine-type d-Ala-d-Ala decarboxylase. CARD-RGI annotated the latter as *vanY*, the only predicted AMR gene across genomes. VanY-mediated vancomycin resistance requires the *vanHAX* operon [101], which is absent from all *P. laudensis* genomes, indicating *vanY* alone is unlikely to confer resistance. The uGI cluster 16, present in all 20 MSC strains, encodes Mg/Co transporters and a *vanZ*-like gene. Unlike *vanY, vanZ* confers teicoplanin (not vancomycin) resistance in *Enterococcus faecalis* by reducing antibiotic binding, independently of *vanHAX* [102,103].

**Fig. 6.**
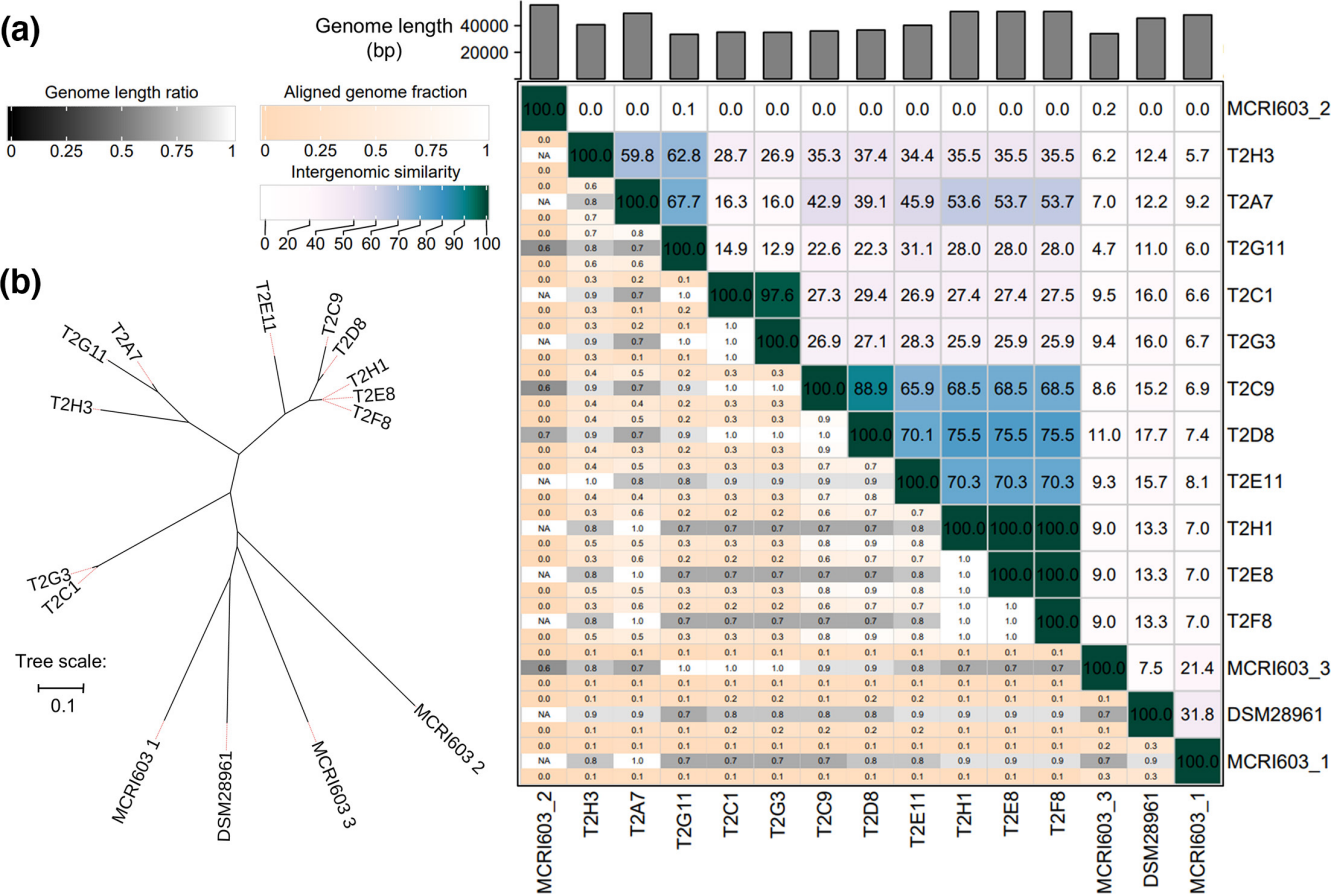
(**a**) Genome lengths, similarity and alignments of the intact prophages identified in *P. laudensis*. (**b**) Unrooted phylogenetic tree based on the proteome of the prophages.

Prophages were predicted in all the *P. laudensis* strains, in agreement with previous findings in *L. lactis* and *L. cremoris* [69]. After analysis with Phastest complemented with manual curation, 13 of the 22 strains were predicted to carry intact prophages, i.e. prophages that still conserve all their basic biological functions and are susceptible to being induced and enter the lytic cycle under determined conditions. Specifically, MCRI-603 harbours three different putative intact prophages, while DSM 28961, T2A7, T2C1, T2C9, T2D8, T2E11, T2E8, T2F8, T2G11, T2G3, T2H1 and T2H3 encompass one each. Among these, T2C1 and T2G3 harbour a similar intact prophage (97.6% similarity), whereas T2H1, T2F8 and T2E8 share a distinct identical prophage. The rest of the strains carry unique putative intact prophages (Fig. 6). Prophage sizes spanned 33,577–55,548 bp for T2G11 and one of the prophages in MCRI-603, respectively (Fig. 6). VIRIDIC-based comparisons indicated substantial diversity: 14 species-level clusters (>95% identity) across 10 genera (>70%). Phylogenetic relationship among the proteomes of the different prophages can be observed in Fig. 6.

Finally, phage satellites were predicted in all *P. laudensis* strains except DSM 28961. Most were classified as PICIs, integrated satellites induced during helper-phage replication that hijack phage structural machinery for packaging and transfer [104]. Several strains carried up to three PICIs (T2C1, T2D8, T2E11, T2E8, T2F8 and T2G11). Additionally, T2G3 encoded a capsid-forming PICI (cf-PICI), which differs from canonical PICIs by encoding its own capsid proteins while relying on helper phages for other virion functions [105]. Genomic coordinates as well as PICI clustering based on their backbone composition can be found in Suppl. Data. A detailed assessment of inducibility, lytic activity, morphology, and formal taxonomic assignment of prophages and satellites is beyond the scope of this study and remains to be investigated.

### Phage defence systems

In response to phage pressure, bacteria have developed an array of defence systems to protect themselves. Bacteria possess a variable number of antiphage systems [106], which can occupy substantial genomic space (e.g. ~2.5% of the genome in *Streptococcus thermophilus*) [107] and are frequently MGE-encoded, promoting rapid acquisition and spread [67,79].

**Fig. 7.**
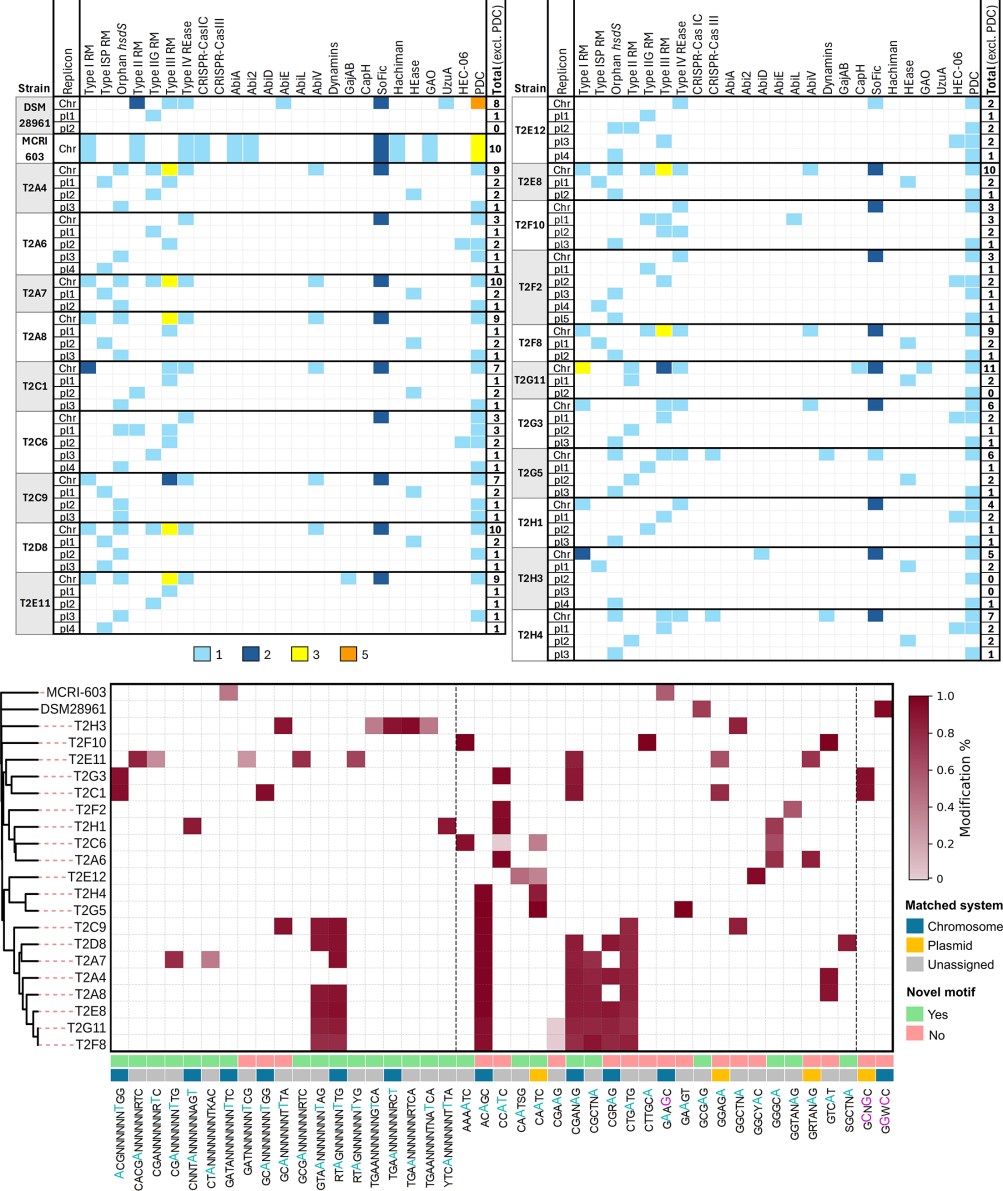
Distribution of (**a**) phage defence systems detected by PADLOC and DefenseFinder in the *P. laudensis* strains and (**b**) methylated motifs. The methylated bases are indicated in the figure in blue (6mA) and pink (5mC).

Using both PADLOC and DefenseFinder, 238 defence systems were identified across the *P. laudensis* genomes (mean 10.82 per strain), of which 151 were chromosomal (63.45%) and 87 plasmid-borne (36.55%). System counts ranged from 6 (T2E12) to 12 (T2G11). PADLOC also predicted 60 phage-defence candidates (PDCs). Of 65 plasmids, 61 encoded ≥1 defence system (rising to 63 when PDCs are included) (Fig. 7), whereas approximately half of chromosomal systems occurred within GIs. Overall, ~68% of the defensome is MGE-encoded, comparable to the 59.6% reported for dairy *L. lactis/cremoris* [27], underscoring the central role of MGEs in phage defence.

We detected 22 antiphage system types, including RM, abortive infection (Abi), Clustered Regularly Interspaced Short Palindromic Repeats and CRISPR-associated proteins (CRISPR-Cas), dynamins, Gabija, CapH, Standalone Fic-domain proteins (SoFic), HEases, Hachiman, GAO19, UzuA and HEC-06 (Fig. 7).

RM systems comprise a sequence-specific recognition module with a DNA MTase, which marks host DNA at specific motifs by attaching a methyl group, and a restriction endonuclease (REase), which restricts the same motifs when unmethylated [108]. These are the most widespread antiphage systems among bacteria [106,109]. In congruence with this general observation, RM are also observed to be the most common defence systems in *P. laudensis*, where we identified 121 RM systems distributed among all the studied strains (5.5 per genome on average). The most abundant RM systems were type III (*n*=44), followed by I (*n*=29, including also ISP), II (*n*=26, including also IIG) and IV (*n*=22), in contrast to recent findings in *L. lactis* and *L. cremoris*, in which type I systems dominated, representing~67% of the total [27]. Nearly all type I and type IV RM systems and ~68% type III systems were chromosomally encoded. Conversely, the totality of type ISP and ~80% of the type II RM systems (including IIG) were plasmid-borne. Orphan *hsdS* genes responsible for type I systems specificity occurred both in plasmids and within the chromosome, predominantly in GIs (Fig. 3). Orphan *hsdS* genes capable of recombination with other type I RM systems encoded either in plasmids or within the chromosome have been reported in *Lactococcus*. Such recombination enhanced phage resistance through increasing the restriction specificity, i.e. the DNA motifs detected and restricted by the system [27,110,112]. The abundance of these genes suggests that the same mechanism may occur in *P. laudensis*. Altogether, RM systems, including orphan *hsdS*, represent 63.87% of the total defensome. Other methylation-based defence systems such as BREX or DISARM were not identified. SoFic AMPylases, i.e. with the ability of ligating AMP to proteins [113,114], were the only other system found to be also present in all *P. laudensis* strains, consistent with their broad distribution across bacteria [106,109].

Abi systems, a diverse group of antiphage systems common in *Lactococcus* that trigger cell dormancy or self-induced cell death upon phage infection [115], were identified in 13 *P*. *laudensis* strains. AbiV was the most common Abi system (present in nine strains), whereas AbiD, AbiE and AbiL were found in single strains in T2H3, DSM 28961 and T2F10, respectively (Fig. 7a). Only AbiL was plasmid-encoded (T2F10 plasmid 1). AbiE (comprising AbiEi/AbiEii) was chromosomally encoded in DSM 28961, as previously reported for lactococci, whereas AbiV and AbiD, typically plasmid-borne [79], were instead chromosomally, and concretely, GI-encoded. Specifically, AbiV within ICEs 1–7 and AbiD as the terminal gene of the intact prophage in T2H3.

Despite being widespread antiphage systems in bacteria, CRISPR-Cas systems are not commonly encountered in lactococci [26,106, 116], where a single instance has been reported: a type III-A CRISPR-Cas system in the conjugative plasmid pKLM in *L. lactis* DGCC7167 [117]. Notably, three *P. laudensis* strains, i.e. MCRI-603, T2H4 and T2G5, harbour one CRISPR-Cas system each. Specifically, MCRI-603 encodes a type I-C system within a chromosomal region initially not predicted as a GI (positions 1,390,982 to 1,397,492 bp), but later detected as an HGT region by Alien Hunter, and T2H4 and T2G5 encompassed a type III-A system in the ICE cluster 44. The latter comprises a defence island also encoding the dynamins LeoABC [106,118] and a toxin-antitoxin system. Similar to *L. lactis* and *L. cremoris* [27], defence islands do not appear to be a common feature in *P. laudensis*. T2G5 and T2H4 shared the same spacers and repeat sequences. blastn revealed 100% homology between the spacer-11 and the intact prophage from T2A7, indicating acquired immunity. Furthermore, the repeat sequence (5′-AAATACAACCGCTCCTCGATAAAAGGGGACGAGAAC-3′) was identical to that of the type III-A systems of *L. lactis* DGCC7167, *P. raffinolactis* Lr_19_14, Lr_19_5 and APC3967 (accession numbers JX524189.1, NZ_CP047628.1, NZ_CP047616.1 and NZ_CP147857.1, respectively). We confirmed that the *P. raffinolactis* strains also encoded their type III-A systems within the chromosome, specifically within MGEs.

Finally, we manually surveyed the *P. laudensis* genomes to search for defence systems not included in the PADLOC/DefenseFinder databases, particularly the novel lactococcal plasmid-encoded systems Rhea, Aristaios, Kamadhenu, Fliodhais-2, Audmula, Rugutis and Hesat [67], as well as Sie proteins encoded within prophages [68]. BLASTP analysis revealed the presence of a Rhea system (~100% similarity) in the AA218 plasmid carried by T2A6, T2E11 and T2F2, the only among the novel systems providing resistance to more than one phage, i.e. P335 and TP-901 [67]. Additionally, blastp revealed 60% similarity between a gene encoded in AA698 plasmid in T2H3 and the Hesat system. Sie proteins were encountered encoded between the integrase and the repressor genes of the intact prophages of DSM 28961, T2D8, T2E8, T2F8, T2H1 and the MCRI-603 prophages 1 and 3 (GIs 11 and 46, respectively; Fig. 3).

### Pan-methylome of *P. laudensis*

MTases can occur either as components of RM systems, paired with a cognate REase, or as orphan MTases lacking an associated REase. Depending on the modified base and position of the methyl group, bacterial DNA methylation is classified as N4-methylcytosine (4mC), 5-methylcytosine (5mC) or N6-methyladenine (6mA). Beyond protecting DNA from REases, methylation contributes in some bacteria to regulation of DNA replication and cell cycle [119,120], DNA mismatch repair [121] and epigenetic control of gene expression and phenotypic differentiation [122,125].

The *P. laudensis* pan-methylome comprises 43 distinct methylated motifs, of which 25 were novel and not present in the REBASE database [73]. This is particularly marked in bipartite motifs typical from canonical type I systems, where 16 of the 19 motifs were novel. The methylome is strongly dominated by 6mA, with only two motifs corresponding to 5mC, and no 4mC was detected. Such 6mA predominance is consistent with previous work in the dairy LAB *S. thermophilus*, where all detected methylation was 6mA [107] and with broader estimates indicating that 6mA accounts for ~75% of bacterial DNA methylation [126].

Most motifs were short, non-palindromic sequences typical of type III, some subtypes of type II and type ISP RM systems (61.76%), followed by long bipartite motif characteristic of most type I systems (32.35%). Only two canonical short palindromic type II motifs (5.88%) were observed, corresponding to the two 5mC modifications (Fig. 7b). In total, 15 motifs could be confidently assigned to specific RM systems in the genome (Fig. 7b, Table S3)

Remarkably, methylation patterns were highly strain specific: except for T2G11 and T2F8, which shared identical profiles, each strain exhibited a unique motif repertoire (Fig. 7b). Intra-species variation has been reported in other bacteria and has led to the proposal that DNA methylation can be exploited for below-species-level binning in metagenomic datasets [127,128]. This motif variability appears to be particularly pronounced in the dairy MSC *P. laudensis* strains, where MTases encoded on MGEs contribute to remarkable methylome diversification even among closely related isolates from the same source.

We also identified 74 orphan MTases across *P. laudensis* genomes (~3.5 per strain), all located within chromosomal MGEs, predominantly in prophages but also other GIs. Phages are known to methylate their own DNA to evade host RM systems during transfer [129]. All orphan MTases were predicted to target type II short palindromic motifs, the most common class for this MTase type [126]. However, such motifs were not detectably methylated in most strains, indicating that the corresponding orphan MTases are inactive (non-functional) once integrated into the *P. laudensis* chromosome. Only T2C1, T2G3 and DSM 28961 showed type II methylated motifs (Fig. 7b), and in all three cases, these were linked to active type II RM systems rather than to orphan MTases.

## Discussion

*P. laudensis* is an emerging dairy-associated species that is attracting increased research attention [15]. It has been detected in raw bovine milk [4], kale [28] and dairy MSC [29]. Its occurrence across food-related niches, together with the reported *β*-amyloid-reducing activity of strain MCRI-603 in *Caenorhabditis elegans* [28], underscores the need for a comprehensive genomic characterization of this species.

In this study, we assembled the complete genome of the plant isolate MCRI-603 and compared it with the milk isolate DSM 28961 and 20 strains from a Danish MSC. Consistent with this limited geographic sampling, the *P. laudensis* pangenome remains open, indicating that additional gene diversity is likely to be recovered as strains from additional geographical regions and ecological niches are included. Comparative analyses, including COG profiling, revealed niche-associated differences, particularly in genes linked to the mobilome and to carbohydrate, amino acid and lipid transport and metabolism, similar to previous findings for *L. lactis* and *L. cremoris* [24].

Detailed analysis of the *P. laudensis* mobilome revealed a niche-associated gradient: dairy starter strains harboured the highest number of transposases, pseudogenes, plasmids, and GIs, whereas the plant isolate MCRI-603 carried almost no IS elements, very few pseudogenes, no plasmids and the lowest number of GIs. DSM 28961 occupied an intermediate position: it was more similar to MCRI-603 than to the MSC strains in terms of synteny, ANI, pangenome and COG profiles yet already showed substantial genome decay (established by the quantity of transposases and pseudogenes) and plasmid-encoded niche traits such as the *lac* operon. Altogether, these observations are consistent with a plant-associated origin for *P. laudensis* and a subsequent dairy adaptation via reductive evolution and progressive MGE acquisition, paralleling evolutionary models proposed for *L. lactis* [2]. Conceptually, this fits with milk as an ecological intermediate between plant material (consumed by cows) and dairy starter cultures, originally developed from continued back-slopping of initially spontaneously fermented milk products [130]. Additional isolates from diverse environments will be required to consolidate these findings.

The mobilome of the dairy-associated *P. laudensis* revealed the presence of positive dairy-relevant traits in both plasmids and chromosomal islands, including the *eps* operon for EPS biosynthesis [32], plasmid-borne transposon-encoded *lac* operons, *opp* genes for transport systems for oligopeptides and an array of antiphage systems. Interestingly, the *eps* and *opp* genes and some defence systems such as CRISPR-Cas have been encountered within GIs in *P. laudensis*, in contrast to dairy *Lactococcus* where these are most commonly encoded in plasmids [26,117, 131], suggesting that dairy *Pseudolactococcus* are more prone to the acquisition of adaptive traits by IEs.

It is noteworthy that several plasmid clusters are shared among dairy *P. laudensis* strains from the same MSC, despite the apparent absence of canonical conjugative plasmids. The identified ICEs encode a complete conjugation machinery, but no OriT could be predicted and actual OriT motifs remain uncharacterized [99]. In line with this, our data does not provide direct evidence that *P. laudensis* plasmids are (co-)mobilized via these ICEs. One possible scenario is that mobilizable *P. laudensis* plasmids could have been mobilized by conjugative elements from other community members with compatible OriT regions [89], effectively hijacking their conjugation systems. Alternatively, plasmid dissemination may occur via phage-mediated transduction. All strains carried prophages or prophage remnants, and DSM 28961, MCRI-603 and 11 MSC isolates harboured at least 1 intact prophage. Future studies examining the inducibility of intact prophages and testing their capacity to pack and transfer plasmids, as well as ICE-mediated transfer [99], will be essential to elucidate the predominant HGT routes in *P. laudensis*. Despite their potential role in HGT, prophages are considered industrially relevant liabilities because induction may trigger cell lysis and contribute to fermentation delays or failures; therefore, assessing prophage inducibility is essential for evaluating the suitability of *P. laudensis* strains for starter culture applications. Genomic analysis revealed a diverse defensome in *P. laudensis* and roughly two-thirds of identified antiphage systems encoded on plasmids or other MGEs, including prophages, promoting strain-level differences. This is in line with observations in *L. lactis* and *L. cremoris* [26,27] and underscores the central role of the mobilome in shaping phage resistance. Notably, CRISPR-Cas systems, specifically types III-A and I-C, were more frequently detected in *P. laudensis* (and *P. raffinolactis*) than in *L. lactis*/*cremoris*, where there is only one instance of CRISPR-Cas in a conjugative plasmid in *L. lactis* DGCC7167 [117]. The complete conservation of type III-A repeat sequences across *P. laudensis*, *P. raffinolactis* and *L. lactis* suggests an ancestral CRISPR origin that has disseminated via MGEs beyond plasmids, adding a new dimension to our understanding of CRISPR-Cas evolution within dairy LAB.

RM systems constituted the most abundant antiphage systems, consistent with dairy *Lactococcus;* however, the *P. laudensis* defensome is dominated by type III RM systems, in contrast to type I systems as in *L. lactis/cremoris* [27]. Our analysis showed that the *P. laudensis* methylome is heavily dominated by 6mA methylation of non-palindromic short motifs, closely followed by bipartite motifs, for which not only complete type I systems but also orphan HsdS surely account. Strikingly, the isolates presented nearly exclusive strain-specific patterns, reflecting evolutionary adaptation to phage pressure and likely having also an influence on epigenetic regulation [132], although functional consequences remain to be tested. Strain-level methylome variability has been reported for dairy *S. thermophilus* and *L. lactis/cremoris* [27,107], but our dataset is distinctive in that all MSC *P. laudensis* isolates originate from a single culture yet still display differentiated methylation profiles. This underscores both the richness of RM systems within a single dairy community and the potential utility of methylation patterns as (i) selectable traits for defined starter culture design and (ii) high-resolution markers for strain discrimination and improved metagenomic binning in dairy microbiomes. Such applications are enabled by PacBio sequencing or Oxford Nanopore sequencing coupled with methylation-aware tools such as Nanomotif [71]. Overall, DNA methylation could help deepen our understanding of the complexity of microbial dairy communities.

The presence of numerous orphan MTases within prophages and other GIs also requires further attention. Although these appeared inactive based on the absence of their predicted type II methylated motifs, MGE-encoded MTases can be functional after integration and may influence host phenotypes through epigenetic regulation, as reported in other bacteria [133]. Future investigations could elucidate if/when these systems become functional and their implications in the *P. laudensis* phenotype, especially in industrially relevant traits.

In addition, uncharacterized PDCs were observed in all strains' genomes and associated with both plasmid and chromosomal content, including GIs. This highlights the possible acquisition of novel anti-phage systems that may add to the defence arsenal of *P. laudensis*. The functionality of such candidate systems remains to be elucidated. Similarly, the genomic context of anti-phage systems in relation to specific MGE subclasses will be relevant to pursue in future studies, especially as additional *P. laudensis* genomes become available from plant-associated and other ecological niches. Examining the distribution of defence system types across prophages, ICEs, IMEs, IEs and uGIs, together with functional assays, will help clarify the biological significance of these MGEs and their roles in phage resistance, niche adaptation and the evolution of *P. laudensis* in dairy and potential non-dairy environments.

In conclusion, this study reveals a complex genomic and epigenomic landscape in *P. laudensis*, in which genome decay and MGEs jointly shape evolution and niche adaptation. We provide evidence consistent with a plant-associated origin and subsequent dairy specialization via progressive acquisition of plasmids and IEs encoding key traits for milk utilization and phage defence. The marked reliance on GIs for functions that are typically plasmid-borne in *Lactococcus*, together with the highly diversified, strain-specific methylomes observed within a single starter culture, underscores the distinctive evolutionary trajectory of *Pseudolactococcus*. This work represents the first comparative genomic analysis of the genus and establishes a foundation for future studies linking these genomic and epigenomic features to concrete technological, ecological and potential health-related phenotypes in *P. laudensis* and related species.

## Supplementary material

10.1099/mgen.0.001779 Supplementary Material 1.

10.1099/mgen.0.001779 Supplementary Material 2.

## Abbreviations

ANI: average nucleotide identity
blastp: Basic Local Alignment Search Tool
CDS: coding sequence
COG: Clusters of Orthologous Groups
CRISPR-Cas: clustered regularly interspaced short palindromic repeats and CRISPR-associated proteins
EPS: exopolysaccharide
GI: genomic island
HGT: horizontal gene transfer
ICE: integrative and conjugative element
IE: integrative element
IME: integrative and mobilizable element
IS: insertion sequence
LAB: lactic acid bacteria
MGE: mobile genetic element
MSC: mesophilic starter culture
MTase: methyltransferase
OriT: origin of transfer
PDC: phage-defence candidate
PICI: phage-inducible chromosomal island
RM: restriction–modification
Sie: superinfection exclusion
T4CP: type IV coupling protein
T4SS: type IV secretion systems
uGI: unidentified genomic island
