# Supplementary material for: Comparative genomics and methylome profiling of Pseudolactococcus laudensis reveal signatures of niche adaptation and strain-level variation in mobile genetic elements and phage defence
Source: Microb Genom. 2026 Jul 6;12(7):001779. doi: 10.1099/mgen.0.001779 (PMC13335696; doi:10.1099/mgen.0.001779)
Supplement: Supplementary Material 1. [file mgen-12-01779-s001.pdf]

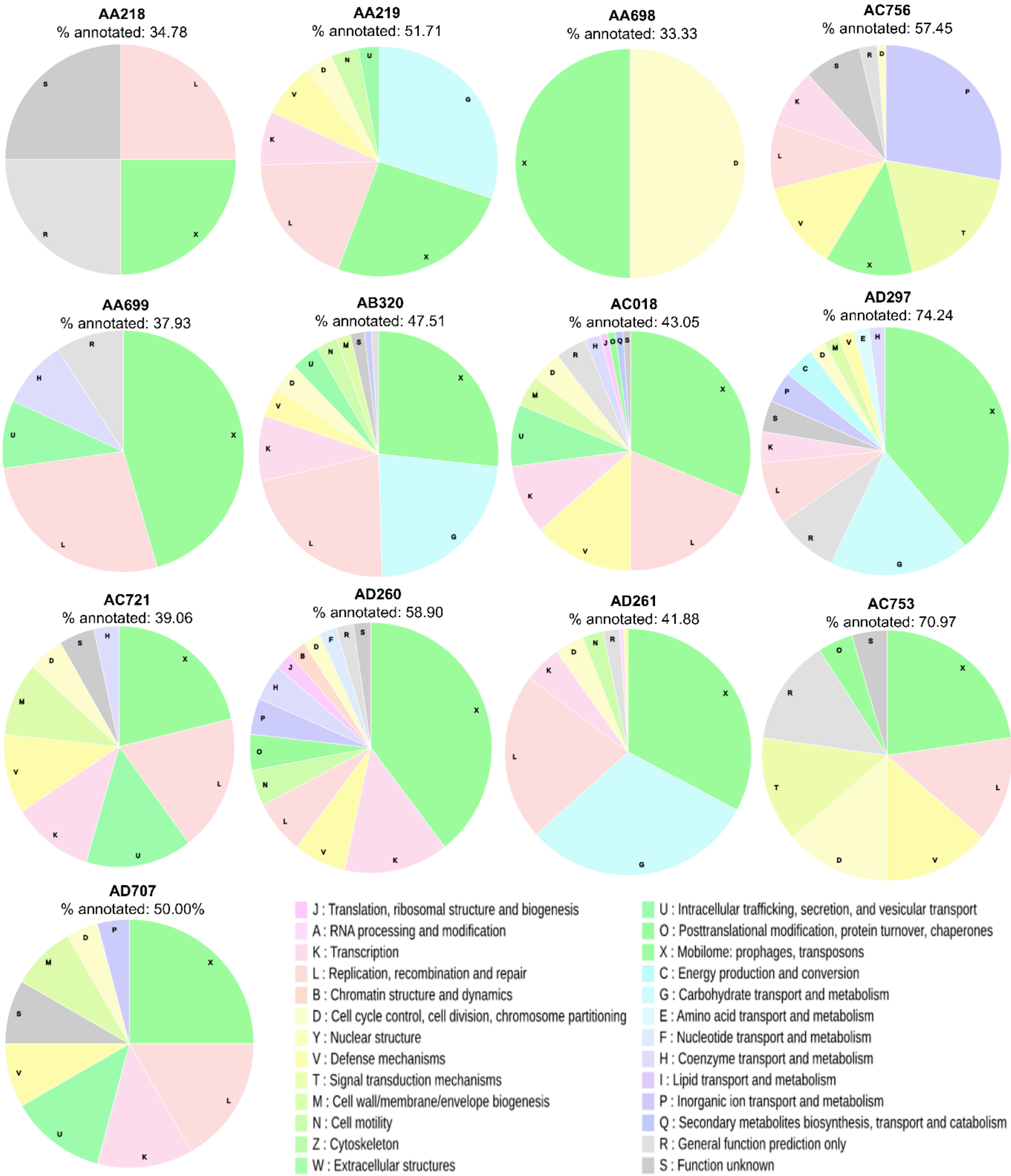

Suppl. Figure S1. COGs per plasmid type (primary cluster ID). Percentages under the plasmid type represent the % of genes for which a COG category could be assigned. In plasmid type AB321 no COG could be annotated.

Supplementary table S1. Assembly quality statistics

Strain	Completeness (%)	Contamination (%)	Strain heterogeneity (%)
DSM28961	97.61	0.25	0
MCRI-603	98.11	0.94	0
T2A4	98.11	0.82	0
T2A6	98.11	0.82	0
T2A7	97.92	1.01	0
T2A8	98.11	0.82	0
T2C1	97.92	0.82	0
T2C6	97.36	0.82	0
T2C9	97.92	0.82	0
T2D8	97.55	0.82	0
T2E11	98.11	0.82	0
T2E12	97.74	0.82	0
T2E8	98.11	0.82	0
T2F10	98.11	0.82	0
T2F2	98.11	0.82	0
T2F8	98.11	0.82	0
T2G11	98.11	1.19	0
T2G3	98.11	0.82	0
T2G5	98.11	0.82	0
T2H1	98.11	0.82	0
T2H3	97.92	1.07	0
T2H4	97.74	0.82	0

Supplementary table S2. Detailed Plasmid mobility genes and annotated genes

strain code	contig	size (bp)	OriT position (bp)	OriT type	Relaxase position (bp)	Relaxase type	T4CP position (bp)	T4SS position (bp)	Primary cluster id	Cluster COUNT	Bakta Annotated genes
T2A4	plasmid 1	64715	-	-	041704..42927	-	-	-	AC018	4	repB, copG, tnp, prgI
	plasmid 2	37446	-	-	015470..15937	-	-	-	AD261	11	repB, copG, tnp(x5), ltrA, mobA, lacXGEFDCBAR
	plasmid 3	13464	9968..10104	MOBP	10553..11785	MOBP	-	-	AC756	20	repB, uspA
T2A6	plasmid 1	39446	-	-	20552..21019	-	-	-	AA219	5	repB, parA, tnp, ltrA, mobA, tnp, lacX, lacG, lacE, lacF, lacD, lacC, lacB, lacA, lacR, pinR, umuC, rapA
	plasmid 2	48990	-	-	27656..28126	-	-	36075..42973	AC721	8	repB, parA, rapA, mod, tnp, ltrA, mobA, traG, herA, prgI
	plasmid 3	13472	9976..10112	MOBP	10561..11793	MOBP	-	-	AC756	20	repB, repX, uspA, mntH, mobC, mobA, mobB
	plasmid 4	10522	9418..9554	MOBP	-	-	-	-	AA218	4	repB, repX
T2A7	plasmid 1	37446	-	-	015470..15937	-	-	-	AD261	11	repB, copG, tnp, ltrA, mobA, lacXGEFDCBAR
	plasmid 2	13464	9968..10104	MOBP	10553..11785	MOBP	-	-	AC756	20	repB, uspA
T2A8	plasmid 1	51433	-	-	027563..28786	-	-	-	AC018	4	repB, copG, mPH1, tnp, prgI
	plasmid 2	37446	-	-	015470..15937	-	-	-	AD261	11	repB, copG, tnp(x5), ltrA, mobA, lacXGEFDCBAR
	plasmid 3	13464	9968..10104	MOBP	10553..11785	MOBP	-	-	AC756	20	repB, uspA
T2C1	plasmid 1	46533	-	-	26780..27247	-	-	-	AB320	5	repB, parA, tnp(x5), mobA, lacXGEFDCBAR
	plasmid 2	37656	-	-	18763..19230	-	-	-	AD261	11	repB, copG, tnp(x3), ltrA, mobA, lacXGEFDCBAR
	plasmid 3	13100	9606..9742	MOBP	010190..11422	MOBP	-	-	AC756	20	repB, uspA
T2C6	plasmid 1	54009	-	-	030749..31222	-	-	-	AB320	5	repB, tnp(x3), alwI, ltrA, mobA
	plasmid 2	48988	-	-	27655..28125	-	-	36074..42972	AC721	8	repB, bcsQ, mPH1, mod, tnp, ltrA, mobA, traG, herA, prgI
	plasmid 3	39446	-	-	20552..21019	-	-	-	AA219	5	repB, parA, tnp(x2), ltrA, mobA, lacXGEFDCBAR
	plasmid 4	13472	9976..10112	MOBP	10561..11793	MOBP	-	-	AC756	20	repB, uspA
T2C9	plasmid 1	37446	-	-	015470..15937	-	-	-	AD261	11	repB, copG, tnp(x5), ltrA, mobA, lacXGEFDCBAR
	plasmid 2	8397	4954..5090	MOBP	5539..6771	MOBP	-	-	AC753	3	repB
	plasmid 3	13464	9968..10104	MOBP	10553..11785	MOBP	-	-	AC756	20	repB, uspA
T2D8	plasmid 1	37446	-	-	015470..15937	-	-	-	AD261	11	repB, copG, tnp(x5), mobA, lacXGEFDCBAR
	plasmid 2	13464	9968..10104	MOBP	10553..11785	MOBP	-	-	AC756	20	repB, uspA

	**plasmid 3**	**9642**	8538..8674	MOBP	-	-	-	-	AA218	4	
**T2E11**	**plasmid 1**	**44109**	-	-	25216..25683	-	-	-	AD261	11	repB, copG, tnp(x3), ltrA, mobA, lacXGEFDCBAR
	**plasmid 2**	**26527**	-	-	04130..5353	-	-	-	AA699	1	repB, tnp, copG
	**plasmid 3**	**13463**	9968..10104	MOBP	10553..11044	MOBP	-	-	AC756	20	repB, uspA
	**plasmid 4**	**10522**	9418..9554	MOBP	-	-	-	-	AA218	4	
**T2E12**	**plasmid 1**	**39445**	-	-	20552..21019	-	-	-	AA219	5	repB, parA, tnp, ltrA, mobA, tnp, lacX, lacG, lacE, lacF, lacD, lacC, lacB, lacA, lacR
	**plasmid 2**	**46309**	-	-	023049..23522	-	-	-	AD707	1	repB, cadDX, tnp(x2), alwI, ltrA, mobA
	**plasmid 3**	**43717**	-	-	-	-	-	30803..37701	AC721	8	repB, bcsQ, mPH1, mod, tnp, traG, herA, prgI
	**plasmid 4**	**13471**	9976..10112	MOBP	10561..11793	MOBP	-	-	AC756	20	repB, uspA
**T2E8**	**plasmid 1**	**37446**	-	-	15470..15937	-	-	-	AD261	11	repB, copG, tnp(x5), ltrA, mobA, lacXGEFDCBAR
	**plasmid 2**	**13464**	9968..10104	MOBP	10553..11044	MOBP	-	-	AC756	20	repB, uspA
**T2F10**	**plasmid 1**	**71484**	-	-	47609..48832	-	-	-	AC018	4	repB, copG, tnp(x3), copG, prgI
	**plasmid 2**	**47779**	-	-	25846..26313	-	-	34865..41763	AC721	8	repB, bcsQ, mcrC, tnp(x2), ltrA, mobA, traG, herA, prgI
	**plasmid 3**	**13471**	9976..10112	MOBP	10561..11793	MOBP	-	-	AC756	20	repB, uspA
**T2F2**	**plasmid 1**	**39447**	-	-	20553..21020	-	-	-	AA219	5	repB, parA, tnp, ltrA, mobA, tnp, lacX, lacG, lacE, lacF, lacD, lacC, lacB, lacA, lacR
	**plasmid 2**	**48990**	-	-	27704..28174	-	-	36123..43021	AC721	8	repB, bcsQ, mPH1, mod, tnp, ltrA, mobA, traG, herA, prgI
	**plasmid 3**	**13472**	9976..10112	MOBP	10561..11793	MOBP	-	-	AC756	20	repB, uspA
	**plasmid 4**	**10522**	9418..9554	MOBP	-	-	-	-	AA218	4	
	**plasmid 5**	**10140**	6697..6833	MOBP	7282..8514	MOBP	-	-	AC753	3	repB, tnp(x2), ebhA, ecsC
**T2F8**	**plasmid 1**	**37446**	-	-	015470..15937	-	-	-	AD261	11	repB, copG, tnp(x5), ltrA, mobA, lacXGEFDCBAR
	**plasmid 2**	**13463**	9968..10104	MOBP	10552..11043	MOBP	-	-	AC756	20	repB, uspA
**T2G11**	**plasmid 1**	**31972**	-	-	13111..13578	-	-	-	AB320	5	repB, parA, tnp(x3), ltrA, mobA, lacXGEFDCBAR
	**plasmid 2**	**13819**	10323..10459	MOBP	10908..12140	MOBP	-	-	AC756	20	repB, tnp, uspA
**T2G3**	**plasmid 1**	**52898**	-	-	-	-	-	-	AC018	4	copG, mod, tnp(x3), prgI
	**plasmid 2**	**37658**	-	-	-	-	-	-	AD261	11	repB, copG, tnp(x3), ltrA, mobA, lacXGEFDCBAR
	**plasmid 3**	**13102**	-	MOBP	-	MOBP	-	-	AC756	20	repB, uspA
**T2G5**	**plasmid 1**	**40878**	-	-	-	-	-	-	AC721	8	repB, traG, herA, prgI
	**plasmid 2**	**36168**	-	-	-	-	-	-	AB320	5	repB, parA, cadD, tnp, alwI (Rease), tnp, ltrA, mobA, tnp, lacXGEFDCBAR
	**plasmid 3**	**17931**	-	MOBP	-	MOBP	-	-	AC756	20	repB, ebhA, tnp, corA, uspA

**T2H1**	**plasmid 1**	**48990**	-	-	-	-	-	-	AC721	8	repB, bcsQ, mPH1, mod, tnp, ltrA, mobA, traG, herA, prgI
	**plasmid 2**	**39446**	-	-	-	-	-	-	AA219	5	repB, parA, tnp, ltrA, mobA, tnp, lacXGEFDCBAR
	**plasmid 3**	**13472**	-	MOBP	-	MOBP	-	-	AC756	20	repB, uspA
**T2H3**	**plasmid 1**	**37512**	-	-	-	-	-	-	AD261	11	repB, copG, tnp, matR, mobA, tnp, lacXGEFDCBAR
	**plasmid 2**	**16897**	-	MOBP	-	MOBP	-	-	AC756	20	repB(x2), uspA, tnp
	**plasmid 3**	**5140**	-	-	-	-	-	-	AA698	1	
	**plasmid 4**	**8398**	-	MOBP	-	MOBP	-	-	AC753	3	repB
**T2H4**	**plasmid 1**	**49022**	-	-	-	-	-	-	AC721	8	repB, parA, mod, ltrA, mobA, traG, herA, tnp, prgI, dbpA
	**plasmid 2**	**36168**	-	-	-	-	-	-	AB320	5	repB, parA, cadD, tnp, alwI (Rease), tnp, ltrA, mobA, tnp, lacXGEFDCBAR
	**plasmid 3**	**17932**	-	MOBP	-	MOBP	-	-	AC756	20	repB, ebhA, tnp, corA, uspA
**DSM 28961**	**plasmid 1**	**61602**	-	-	-	-	-	-	AD260	1	copG, tnp(x8), clpL, cadX, cadD, cadA, nsr, sacB, hicA, ltrA, mobA
	**plasmid 2**	**52635**	-	-	-	-	-	-	AD297	1	tnp (x4), ltrA, mobA

Supplementary table S3. Methylated motifs, predicted responsible enzymes, and novelty

Strain	Enzymes	DNA	Locus	Type	System Length	Motif	type	Count	Unique	New on REBASE?	Genuine	% Detected	Notes
DSM 28961	Unassigned	-	-	II		GCGAG	non-palindromic	1793	yes	yes	y	70	
DSM 28961	M.Pla28961I	chromosome	3395	II	465 aa	GGWCC	palindromic	959	no	no	y	96.5/96.5	
MCRI-603	M.Pla603I	chromosome	6470	I gamma	528 aa	GATANNNNNNNTTC	bipartite	381	yes	yes	y	-/84.8	
MCRI-603	M1.Pla603II	-	5980	II	365 aa	GAAGC	non-palindromic	2437	no	no	y	-	
MCRI-603	M2.Pla603II	-	5985	II gamma	541 aa	GAAGC	non-palindromic	2437	no	no	y	65.6/45.3	
T2A4	M.PlaT2A4III	chromosome	8205	III beta	707 aa	ACAGC	non-palindromic	4302	no	no	y	98.5	
T2A4	M.PlaT2A4I	chromosome	9075	III beta	677 aa	CGANAG	non-palindromic	2790	no	yes	y	87.5	
T2A4	Unassigned	-	-	II	-	CGCTNA	non-palindromic	3604	no	yes	y	82.7	
T2A4	Unassigned	-	-	II	-	CGNAG		6692	no	no	y	1	
T2A4	PlaT2A4II	chromosome	10750	II G,S	1444 aa	CGRAG	non-palindromic	3108	no	no	y	87.5	
T2A4	Unassigned	-	-	II	-	CTGATG	non-palindromic	1976	no	no	y	83.2	
T2A4	Unassigned	-	-	II	-	GTCAT	non-palindromic	6679	no	no	y	92.2	
T2A6	M.PlaT2A6I	plasmid unnamed2	11550	III beta	624 aa	CAATC	non-palindromic	7656	no	yes	y	96.9	
T2A6	Unassigned	-	-	II		GGGCA	non-palindromic	2204	no	yes	y	77.1	
T2A6	Unassigned	-	-	II		GRTANAG	non-palindromic	2958	no	no	y	85	
T2A7	M.PlaT2A7III	chromosome	8770	III beta	707 aa	ACAGC	non-palindromic	4270	no	no	y	97.9	
T2A7	M.PlaT2A7II	chromosome	9660	III beta	662 aa	CGANAG	non-palindromic	2771	no	yes	y	85	
T2A7	Unassigned	-	-	I		CGANNNNNNTTG	bipartite	1067	yes	yes	y	63.4/93.9	
T2A7	Unassigned	-	-	II		CGCTNA	non-palindromic	3622	no	yes	y	77.8	
T2A7	Unassigned	-	-	I		CTANNNNNNNTKAC		428	yes	yes	y	40.9	

T2A7	Unassigned	-	-	II		CTGATG	non-palindromic	1976	no	no	y	80.7
T2A7	M.PlaT2A7I	chromosome	1980	I gamma	531 aa	RTAGNNNNNNTTG	bipartite	739	no	yes	y	90.7/94.0
T2A8	M.PlaT2A8IV	chromosome	8325	III beta	707 aa	ACAGC	non-palindromic	4259	no	no	y	98.2
T2A8	M.PlaT2A8I	chromosome	9205	III beta	677 aa	CGANAG	non-palindromic	2779	no	yes	y	87.3
T2A8	Unassigned	-	-	II		CGCTNA	non-palindromic	3587	no	yes	y	86
T2A8	Unassigned	-	-	II		CTGATG	non-palindromic	1966	no	no	y	83.9
T2A8	Unassigned	-	-	II		GTCAT	non-palindromic	6664	no	no	y	90.8
T2A8	M.PlaT2A8II	-		I	dummy	GTAANNNNNNNTAG	bipartite	193	no	yes	y	89.6/88.1
T2A8	M.PlaT2A8III	chromosome	1820	I gamma	531 aa	RTAGNNNNNNTTG	bipartite	744	no	yes	y	91.1/91.3
T2C1	M.PlaT2C1IV	chromosome	1825	I gamma	531 aa	ACGNNNNNNTGG	bipartite	701	no	yes	y	90.0/96.9
T2C1	M.PlaT2C1I	chromosome	9340	III beta	677 aa	CGANAG	non-palindromic	2754	no	yes	y	89.6
T2C1	M.PlaT2C1III	chromosome	6390	I gamma	531 aa	GCANNNNNNNTGG	bipartite	999	no	no	y	94.6/95.1
T2C1	M.PlaT2C1II	plasmid unnamed2	12170	II	317 aa	GCNGC	palindromic	5606	no	no	y	92.2/92.2
T2C1	M1.PlaT2C1V	-	11905	III	388 aa	GGAGA	non-palindromic	2430	no	no	y	79.3
T2C1	M2.PlaT2C1V	-	11920	III beta	689 aa	GGAGA	non-palindromic	2430	no	no	y	-
T2C6	M1.PlaT2C6I	-	11430	II alpha	327 aa	CCATC	non-palindromic	3265	no	no	y	-
T2C6	M2.PlaT2C6I	-	11435	II alpha	416 aa	CCATC	non-palindromic	3265	no	no	y	-/77.3
T2C6	M.PlaT2C6II	plasmid unnamed2	11685	III beta	624 aa	CAATC		7737	no	yes	y	0.5
T2C6	Unassigned	-	-	II		GGGCA	non-palindromic	2227	no	yes	y	63
T2C6	Unassigned	-	-	II		AAAATC	non-palindromic	6069	no	yes	y	92.1
T2C9	M.PlaT2C9IV	chromosome	8595	III beta	707 aa	ACAGC	non-palindromic	4241	no	no	y	98.3
T2C9	Unassigned	-	-	II		CTGATG	non-palindromic	1951	no	no	y	80.2
T2C9	M.PlaT2C9III	-		I	dummy	GCANNNNNNTTA	bipartite	405	no	no	y	91.6/91.1

T2C9	Unassigned	-	-	II		GGCTNA	non-palindromic	3792	no	no	y	84.9	
T2C9	M.PlaT2C9II	-		I	dummy	GTAANNNNNNNTAG	bipartite	196	no	yes	y	89.8/87.8	
T2C9	M.PlaT2C9I	chromosome	1825	I gamma	531 aa	RTAGNNNNNNTTG	bipartite	745	no	yes	y	91.3/93.0	
T2D8	M.PlaT2D8V	chromosome	8590	III beta	707 aa	ACAGC	non-palindromic	4288	no	no	y	98.1	
T2D8	M.PlaT2D8I	chromosome	9460	III beta	677 aa	CGANAG	non-palindromic	2795	no	yes	y	86.7	
T2D8	PlaT2D8IV	chromosome	11130	II G,S	1444 aa	CGRAG	non-palindromic	3114	no	no	y	89	
T2D8	M.PlaT2D8IV	chromosome	11135	II	243 aa	CGRAG	non-palindromic	3114	no	no	y	-	
T2D8	Unassigned	-	-	II		CTGATG	non-palindromic	1959	no	no	y	82	
T2D8	M.PlaT2D8III	-		I	dummy	GTAANNNNNNNTAG	bipartite	198	no	yes	y	90.4/87.9	
T2D8	M.PlaT2D8II	chromosome	1825	I gamma	531 aa	RTAGNNNNNNTTG	bipartite	752	no	yes	y	90.0/93.6	
T2D8	Unassigned	-	-	II		SGCTNA	non-palindromic	7518	yes	yes	y	86.2	
T2E11	Unassigned	-	-	I		CACGANNNNNNRTC	bipartite	76	yes	yes	y	82.9	
T2E11	M.PlaT2E11I	chromosome	9480	III beta	677 aa	CGANAG	non-palindromic	2768	no	yes	y	84.9	
T2E11	M.PlaT2E11III	-		I	dummy	CGANNNNNNRTC	bipartite	1003	yes	yes	y	-/64.7	
T2E11	Unassigned	-	-	I		GATNNNNNNTCG	bipartite	549	no	no	y	-/53.9	
T2E11	Unassigned	-	-	I		GCGANNNNNNRTC	bipartite	266	yes	yes	y	77.1	
T2E11	M1.PlaT2E11II	-	11815	III	388 aa	GGAGA	non-palindromic	2402	no	no	y	-	
T2E11	M2.PlaT2E11II	-	11825	III beta	689 aa	GGAGA	non-palindromic	2402	no	no	y	61.2	
T2E11	PlaT2E11IV	plasmid unnamed2	12065	II G,S	1572 aa	GRTANAG	non-palindromic	3031	no	no	y	75.1	
T2E11	Unassigned	-	-	I		RTAGNNNNNNTYG	bipartite	1020	yes	yes	y	66.1/68.9	
T2E12	M1.PlaT2E12I	-	11590	II alpha	327 aa	CCATC	non-palindromic	3243	no	no	y	-	
T2E12	M2.PlaT2E12I	-	11595	II alpha	416 aa	CCATC	non-palindromic	3243	no	no	y	-/73.0	
T2E12	Unassigned	-	-	II		CAATSG		3541	yes	yes	y	47.2	CAATSG may be a miscall for CAATC

T2E12	Unassigned	-	-	II		GGCYAC	non-palindromic	802	no	no	y	95.8	
T2E8	M.PlaT2E8V	chromosome	8595	III beta	707 aa	ACAGC	non-palindromic	4280	no	no	y	98.2	
T2E8	M.PlaT2E8I	chromosome	9475	III beta	677 aa	CGANAG	non-palindromic	2777	no	yes	y	86.8	
T2E8	Unassigned	-	-	II		CGCTNA	non-palindromic	3651	no	yes	y	86.4	
T2E8	PlaT2E8II	chromosome	11140	II G,S	1444 aa	CGRAG	non-palindromic	3101	no	no	y	-	
T2E8	M.PlaT2E8II	chromosome	11145	II	243 aa	CGRAG	non-palindromic	3101	no	no	y	89.1	
T2E8	Unassigned	-	-	II		CTGATG	non-palindromic	1959	no	no	y	81.7	
T2E8	M.PlaT2E8III	-		I	dummy	GTAANNNNNNNTAG	bipartite	199	no	yes	y	90.5/88.9	
T2E8	M.PlaT2E8IV	chromosome	1820	I gamma	531 aa	RTAGNNNNNNTTG	bipartite	750	no	yes	y	91.2/94.3	
T2F10	Unassigned	-	-	II		CTTGCA	non-palindromic	1437	no	no	y	93.7	
T2F10	Unassigned	-	-	II		GTCAT	non-palindromic	6614	no	no	y	90.3	M.PlaT2F10ORF11395P may be responsible for GTCAT
T2F10	Unassigned	-	-	II		AAAATC	non-palindromic	6013	no	yes	y	91.6	
T2F2	M.PlaT2F2I	plasmid unnamed2	11595	III beta	624 aa	CAATC	non-palindromic	7656	no	yes	y	94.2	
T2F2	Unassigned	-	-	II		GGTANAG	non-palindromic	1037	yes	yes	y	57.7	
T2F8	M.PlaT2F8III	chromosome	8635	III beta	707 aa	ACAGC	non-palindromic	4280	no	no	y	92.9	
T2F8	M.PlaT2F8I	chromosome	9510	III beta	677 aa	CGANAG	non-palindromic	2775	no	yes	y	78.1	
T2F8	Unassigned	-	-	II		CGCTNA	non-palindromic	3654	no	yes	y	89.9	
T2F8	PlaT2F8II	chromosome	11190	II G,S	1444 aa	CGRAG	non-palindromic	3099	no	no	y	-	
T2F8	M.PlaT2F8II	chromosome	11195	II	243 aa	CGRAG	non-palindromic	3099	no	no	y	80.2	
T2F8	Unassigned	-	-	III beta		CGAAG		2249	no	no	y	1.5	
T2F8	Unassigned	-	-	II		CTGATG	non-palindromic	1964	no	no	y	84.4	
T2F8	M.PlaT2F8IV	-		I	dummy	GTAANNNNNNNTAG	bipartite	198	no	yes	y	84.9/71.2	
T2F8	M.PlaT2F8V	chromosome	1825	I gamma	531 aa	RTAGNNNNNNTTG	bipartite	749	no	yes	y	68.2/88.6	

T2G11	M.PlaT2G11I	chromosome	9645	III beta	623 aa	ACAGC	non-palindromic	4254	no	no	y	92.9	
T2G11	Unassigned	-	-	II		CGANAG	non-palindromic	2758	no	yes	y	82.4	
T2G11	Unassigned	-	-	II		CGCTNA	non-palindromic	3635	no	yes	y	81.2	
T2G11	Unassigned	-	-	II		CGRAG	non-palindromic	3123	no	no	y	83.6	
T2G11	Unassigned	-	-	II		CGAAG		2262	no	no	y	1	
T2G11	Unassigned	-	-	II		CTGATG	non-palindromic	1971	no	no	y	77.5	
T2G11	Unassigned	-	-	I		GTAANNNNNNNTAG	bipartite	194	no	yes	y	85.1/82.6	
T2G11	Unassigned	-	-	I		RTAGNNNNNNTTG	bipartite	749	no	yes	y	84.4/87.8	
T2G3	M.PlaT2G3III	chromosome	1815	I gamma	531 aa	ACGNNNNNNTGG	bipartite	698	no	yes	y	89.1/95.3	
T2G3	M.PlaT2G3I	chromosome	9260	III beta	677 aa	CGANAG	non-palindromic	2771	no	yes	y	88	
T2G3	M.PlaT2G3IV	plasmid unnamed1	11785	III beta	624 aa	CAATC	non-palindromic	7906	no	yes	y	97.2	M.PlaT2G3ORF11785P is almost certainly responsible for CAATC as the same motif and gene is in other Pla strains
T2G3	M.PlaT2G3II	plasmid unnamed2	12050	II	317 aa	GCNGC	palindromic	5601	no	no	y	92.9/92.9	
T2G5	M.PlaT2G5II	chromosome	8625	III beta	707 aa	ACAGC	non-palindromic	4327	no	no	y	96.9	
T2G5	M1.PlaT2G5I	-	12070	II alpha	327 aa	CCATC	non-palindromic	3357	no	no	y	-	
T2G5	M2.PlaT2G5I	-	12075	II alpha	416 aa	CCATC	non-palindromic	3357	no	no	y	96.5/67.9	
T2G5	Unassigned	-	-	II		GAAGT	non-palindromic	5065	no	no	y	94.1	
T2H1	M.PlaT2H1I	chromosome	1830	I gamma	531 aa	CNNTANNNNNNAGT	bipartite	893	yes	yes	y	88.2/87.5	
T2H1	M.PlaT2H1III	plasmid unnamed1	11740	III beta	624 aa	CAATC	non-palindromic	7796	no	yes	y	96.2	M.PlaT2H1ORF11740P is almost certainly responsible for CAATC as the same motif and gene is in other Pla strains.
T2H1	Unassigned	-	-	II		GGGCA	non-palindromic	2247	no	yes	y	71.7	PlaT2H1ORF11935P may be responsible for GGGCA as a very similar gene is in another strain with this motif.

T2H1	M.PlaT2H1II	-		I	dummy	YTCANNNNNNTTTA	bipartite	466	yes	yes	y	89.9/86.3	Dummy enzyme to enter the PacBio methylation data from this system's solitary subunit, recognition sequence confirmed on genome by PacBio via sequence comparison, ONT recognition sequence
T2H3	M.PlaT2H3I	-		I	dummy	GCANNNNNNTTTA	bipartite	416	no	no	y	91.2/89.4	Dummy enzyme to enter the PacBio methylation data from this system's solitary subunit, recognition sequence confirmed on genome by PacBio via sequence comparison, ONT recognition sequence
T2H3	Unassigned	-	-	II		GGCTNA	non-palindromic	3823	no	no	y	84.1	
T2H3	Unassigned	-	-	I		TGAANNNNNNNGTCA	bipartite	186	yes	yes	y	-/80.1	
T2H3	M.PlaT2H3II	chromosome	6670	I gamma	531 aa	TGAANNNNNNNRCT	bipartite	1177	yes	yes	y	93.5/88.0	
T2H3	Unassigned	-	-	I		TGAANNNNNNNRTCA	bipartite	496	yes	yes	y	95	
T2H3	Unassigned	-	-	I		TGAANNNNNTNATCA	bipartite	72	yes	yes	y	-/83.3	
T2H4	M.PlaT2H4II	chromosome	8640	III beta	707 aa	ACAGC	non-palindromic	4346	no	no	y	98.3	
T2H4	M1.PlaT2H4I	-	12110	II alpha	327 aa	CCATC	non-palindromic	3341	no	no	y	-	
T2H4	M2.PlaT2H4I	-	12115	II alpha	416 aa	CCATC	non-palindromic	3341	no	no	y	94.8/75.2	
T2H4	M.PlaT2H4III	plasmid unnamed1	11920	III beta	624 aa	CAATC	non-palindromic	7934	no	yes	y	87.4	
